# An Engineered Biliverdin-Compatible Cyanobacteriochrome Enables a Unique Ultrafast Reversible Photoswitching Pathway

**DOI:** 10.3390/ijms22105252

**Published:** 2021-05-16

**Authors:** Sean R. Tachibana, Longteng Tang, Liangdong Zhu, Yuka Takeda, Keiji Fushimi, Yoshibumi Ueda, Takahiro Nakajima, Yuto Kuwasaki, Moritoshi Sato, Rei Narikawa, Chong Fang

**Affiliations:** 1 Department of Chemistry, Oregon State University, 153 Gilbert Hall, Corvallis, OR 97331-4003, USA; tachibas@oregonstate.edu (S.R.T.); tanglo@oregonstate.edu (L.T.); zhul@oregonstate.edu (L.Z.); 2 Graduate School of Integrated Science and Technology, Shizuoka University, Shizuoka 422-8529, Japan; 0830mon1201@gmail.com (Y.T.); fushimi.keiji@shizuoka.ac.jp (K.F.); narikawa.rei@shizuoka.ac.jp (R.N.); 3 Graduate School of Arts and Sciences, University of Tokyo, Tokyo 153-8902, Japan; yoshibumiueda@gmail.com (Y.U.); ctnaka@mail.ecc.u-tokyo.ac.jp (T.N.); kuwasakiyuto@g.ecc.u-tokyo.ac.jp (Y.K.); moritoshisato@g.ecc.u-tokyo.ac.jp (M.S.)

**Keywords:** far-red/orange cyanobacteriochromes, structure-activity relationships, time-resolved spectroscopy, reversible photoswitching, optogenetics

## Abstract

Cyanobacteriochromes (CBCRs) are promising optogenetic tools for their diverse absorption properties with a single compact cofactor-binding domain. We previously uncovered the ultrafast reversible photoswitching dynamics of a red/green photoreceptor AnPixJg2, which binds phycocyanobilin (PCB) that is unavailable in mammalian cells. Biliverdin (BV) is a mammalian cofactor with a similar structure to PCB but exhibits redder absorption. To improve the AnPixJg2 feasibility in mammalian applications, AnPixJg2_BV4 with only four mutations has been engineered to incorporate BV. Herein, we implemented femtosecond transient absorption (fs-TA) and ground state femtosecond stimulated Raman spectroscopy (GS-FSRS) to uncover transient electronic dynamics on molecular time scales and key structural motions responsible for the photoconversion of AnPixJg2_BV4 with PCB (Bpcb) and BV (Bbv) cofactors in comparison with the parent AnPixJg2 (Apcb). Bpcb adopts the same photoconversion scheme as Apcb, while BV4 mutations create a less bulky environment around the cofactor D ring that promotes a faster twist. The engineered Bbv employs a reversible clockwise/counterclockwise photoswitching that requires a two-step twist on ~5 and 35 picosecond (ps) time scales. The primary forward P_fr_ → P_o_ transition displays equal amplitude weights between the two processes before reaching a conical intersection. In contrast, the primary reverse P_o_ → P_fr_ transition shows a 2:1 weight ratio of the ~35 ps over 5 ps component, implying notable changes to the D-ring-twisting pathway. Moreover, we performed pre-resonance GS-FSRS and quantum calculations to identify the Bbv vibrational marker bands at ~659,797, and 1225 cm^−1^. These modes reveal a stronger H-bonding network around the BV cofactor A ring with BV4 mutations, corroborating the D-ring-dominant reversible photoswitching pathway in the excited state. Implementation of BV4 mutations in other PCB-binding GAF domains like AnPixJg4, AM1_1870g3, and NpF2164g5 could promote similar efficient reversible photoswitching for more directional bioimaging and optogenetic applications, and inspire other bioengineering advances.

## 1. Introduction

Phytochromes, bacteriophytochromes, and cyanobacteriochromes (CBCRs) are among a superfamily of linear tetrapyrrole (bilin chromophore that is metabolically derived from heme) binding photoswitching proteins [1,2,3]. In nature, these proteins act as photosensing and signaling units that allow organisms to detect and respond to light from the UV to near-IR range [4,5,6,7,8]. Previous efforts have engineered these proteins as both non-invasive optogenetic tools [9,10,11] and fluorescent tags in vivo [12,13]. CBCRs have shown to be a versatile tool in optogenetic applications by requiring only a single compact domain for incorporation of the cofactor chromophore, the photosensing unit that can be considered as the active site [3,14]. Recently, we studied a red/green-absorbing (^15*Z*^P_r_/^15*E*^P_g_) hypsochromic CBCR named AnPixJg2 using the wavelength-tunable femtosecond transient absorption (fs-TA) and ground-state femtosecond stimulated Raman spectroscopy (GS-FSRS) [15]. We uncovered the initial ultrafast dynamics as the phycocyanobilin (PCB cofactor in protein matrix) photoswitches from the P_g_ to P_r_ and P_r_ to P_g_ conformers in a reversible manner (controlled by a combination of actinic laser pump pulses and continuous-wave/cw LEDs). The nomenclature of *Z* and *E* refers to the C–D ring methine bridge C15=C16 configuration in the *cis* and *trans* state, respectively. The P_g_ → P_r_ conversion exhibits a more downhill reaction with a ~3 ps lifetime to reach a conical intersection (CI) and relaxes into a Lumi-G state on the ~30 ps time scale. In contrast, the P_r_ → P_g_ conversion shows a longer-lived excited state with ~13 and 217 ps time constants to reach a CI, which takes a much longer time to further relax in the ground state of Lumi-R and beyond.

**Figure 1:**
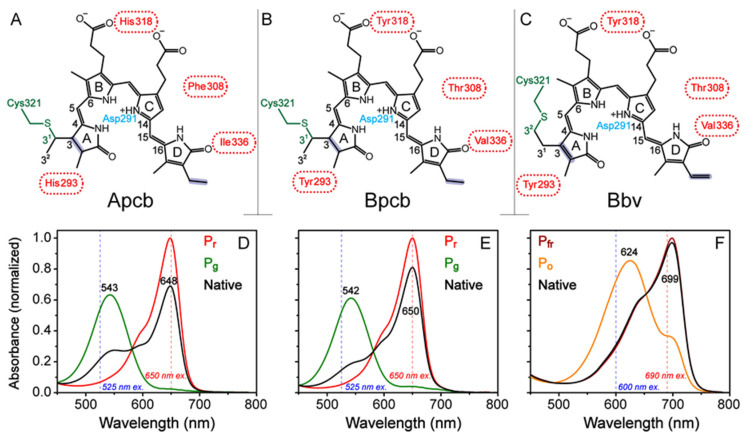
Representative chromophore and surrounding protein residues of (**A**) AnPixJg2_PCB, (**B**) AnPixJg2_BV4_PCB, and (**C**) AnPixJg2_BV4_BV. The associated native/dark-adapted states of ^15*Z*^P_r_, ^15*Z*^P_r_, and ^15*Z*^P_fr_ are depicted. Four residues responsible for the BV incorporation are shown (red) with Cys321 (green) that covalently binds cofactor to protein pocket. Key changes to cofactor conjugation are highlighted by purple shades with Asp291 (cyan) as an important H-bonding partner to A, B, and C rings. The absorption spectra of (**D**) Apcb, (**E**) Bpcb, and (**F**) Bbv are normalized to the reddest-absorbing species. Sample spectra were collected after ambient light, 505 nm LED, 650 nm LED, 600 nm LED, and a tungsten lamp with 650 nm longpass filter irradiation for 5 min for the native (black), P_r_ (red), P_g_ (green), P_fr_ (dark red), and P_o_ (orange) species, respectively. The actinic pump wavelengths for fs-TA experiments are labeled by vertical dashed blue and red lines in each panel.

Although AnPixJg2 has shown to be an efficient photoswitching protein, some major drawbacks involve the lack of PCB in mammalian cells and a green absorbing (^15*E*^P_g_) form outside the optimal biological window, which render it less ideal for mammalian applications. Many protein engineers have drawn inspirations from similar photoswitching proteins such as bacteriophytochromes, which incorporate the biliverdin (BV) cofactor that is intrinsic in mammalian cells. BV also red-shifts the absorption profiles of both conformational states due to a larger electronic conjugation than the PCB molecular framework [16]. Unlike CBCRs, bacteriophytochromes require three domains as a large photosensory core module with global structural preferences for BV incorporation and are typically used as fluorescent probes due to their higher fluorescence quantum yield [17]. In contrast, CBCRs that incorporate PCB as the cofactor exhibit low binding affinity for BV due to the double-bond carbon that cysteine (e.g., Cys321) covalently binds to (see Figure 1A–C). The different binding sites shift the cofactor location in the pocket, which decreases binding and protein expression [18].

To expand the functional space of CBCRs, the canonical AnPixJg2 has been engineered to accept the BV cofactor (AnPixJg2_BV4) with a solved crystal structure (PDB: 5ZOH) [18]. This advance was achieved by mutating four key residues around the cofactor pocket (H293Y, F308T, H318Y, and I336V). The BV incorporation red-shifts the red/green absorption to a far-red/orange absorption profile. The overall structure of AnPixJg2 with PCB cofactor (Apcb for short) and AnPixJg2_BV4 with BV cofactor (Bbv for short) was shown to be quite similar. The major structural difference was identified to be the BV cofactor shifted by ~0.75 Å due to the canonical Cys321 covalently binding at the C3^2^ instead of C3^1^ in PCB (Figure 1A–C). Although shifted in Bbv, five crucial residues responsible for cofactor stability (W289, D291, R301, H322, and Y352) were also shown to be highly conserved in the cofactor vicinity. Of the four mutations (H293Y, F308T, H318Y, and I336V), F308T and I366V were found to be the most crucial for BV incorporation and expression. Both of these residues are located near cofactor C and D rings that reduce steric hindrance upon the BV-acceptable mutations. Besides achieving spatially compact residues, F308T and H318Y both contribute to better stabilize the C ring propionate group through H-bonding. The same BV4 mutations were also demonstrated in several other PCB GAF domains with clearly improved BV-binding efficiencies [18].

Since internal conversion between the spin-allowed electronic states typically occurs on ultrafast time scales that precede fluorescence events on the nanosecond (ns) time scale [19], the tracking of Bbv cofactor ultrafast dynamics could uncover how the BV4 mutations affect the photoswitching pathways in real time. We implemented a systematic study using fs-TA with global analysis, and GS-FSRS with quantum calculations of AnPixJg2_BV4 with PCB cofactor (Bpcb for short), Apcb, and Bbv. These unique CBCR samples allow for a direct comparison of the same cofactor in different pockets (Apcb vs. Bpcb), and different cofactors in the same pocket (Bpcb vs. Bbv). Correlating the effects of the BV4 mutations and BV cofactor on reversible photoswitching pathways with primary electronic and structural events in the non-equilibrium regime could facilitate future rational design of CBCRs, photoswitching proteins with cofactors, and other functional photochromic systems in general.

## 2. Results and Discussions

### 2.1. Steady-State Electronic Spectroscopy with Altered Structural Contexts in CBCR Pockets

In line with our previous study [15], a home-built LED box that fits four 5 mm through-hole LEDs was implemented to keep the samples in their respective conformers for all the spectroscopic experiments. LEDs with center wavelengths at 505, 600, and 650 nm were used to convert CBCRs to the ^15*Z*^P_r_, ^15*Z*^P_fr_, and ^15*E*^P_g_ conformers, respectively, and maintain those species in the sample reservoir during fs-TA and GS-FSRS experiments. To achieve conversion toward the ^15*E*^P_o_ conformer of Bbv, a tungsten lamp with a 650 nm longpass filter was used since our available LEDs did not convert the CBCR sample fast enough. The sample solution was then peristaltically pumped into a flow cell to prevent the LED irradiation from interfering with ultrafast spectroscopic measurements and also allow fresh sample solution to flow continuously, so refreshed samples always reach the laser irradiation spot for spectral data collection. For fs-TA experiments (see detailed results in Section 2.2 below), a 525 and 650 nm actinic pump (~100 fs duration [20]) was used to excite the P_g_ and P_r_ conformers, respectively, of both Apcb and Bpcb (Figure 1D,E). For Bbv, due to the red-shifted ground state absorption profile, a 600 and 690 nm actinic pump (~100 fs duration) was implemented to excite the P_o_ and P_fr_ conformers (15*E* and 15*Z* isomers), respectively (Figure 1F).

The steady-state electronic absorption spectrum of the native CBCR conformer was the first spectrum collected and the sample in buffer solution was allowed to equilibrate under ambient light for 5 min. Afterwards, the converted/target species was generated by specific light irradiation (see Table 1) for 5 min due to accumulated photoswitching from the starting species under a cw-light source [15]. Each spectrum was fitted using Gaussian peaks to identify major peak positions of 648/543, 650/542, and 699/624 nm for the Apcb P_r_/P_g_, Bpcb P_r_/P_g_, and Bbv P_fr_/P_o_ conformers, respectively. Based on the spectra of native conformers, the BV4 mutations seem to promote a more homogeneous ground-state population as evidenced by the predominant P_r_ and P_fr_ forms of Bpcb and Bbv, respectively (Figure 1E,F, black traces). Upon comparison of the P_r_ and P_g_ absorption spectra of Apcb and Bpcb (Figure 1D,E, red and green traces), the BV4 mutations also cause a larger energy difference from 543/648 nm (~2984 cm^−1^) to 542/650 nm (~3066 cm^−1^). More importantly, the extended π-conjugation of Bbv cofactor molecular framework (Figure 1C) improves its π-π stacking with Trp289 [18] that contributes to the notably redder absorption of both P_o_ and P_fr_ states (Figure 1F, orange and dark red traces) than P_g_ and P_r_ states in Apcb as well as Bpcb (Figure 1D,E, green and red traces).

### 2.2. Time-Resolved Electronic Spectroscopy Tracks Reversible Photoswitching of PCB and BV Cofactors in the AnPixJg2_BV4 Pocket

**Figure 2:**
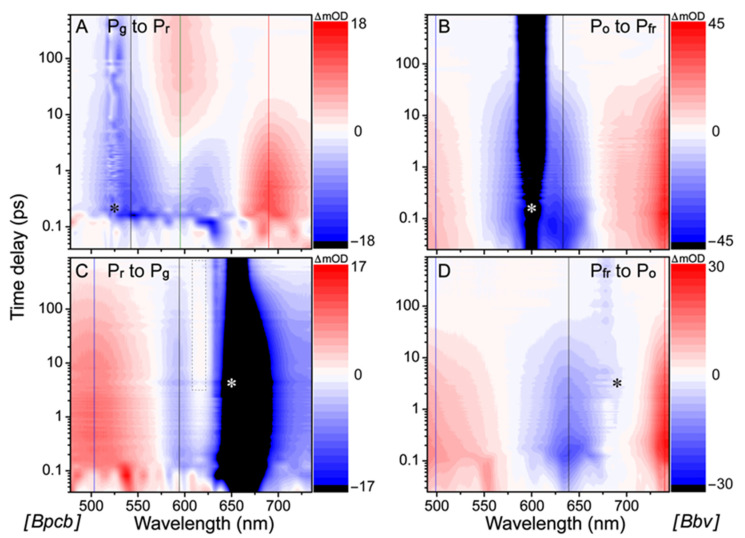
Time-resolved electronic spectra during reversible photoswitching of Bpcb and Bbv in buffer solution. The fs-TA 2D-contour plots of (**A**) Bpcb P_g_ → P_r_, (**C**) Bpcb P_r_ → P_g_, (**B**) Bbv P_o_ → P_fr_, and (**D**) Bbv P_fr_ → P_o_ transitions were collected using a 525 nm actinic pump with 650 nm LEDs, 650 nm actinic pump with 505 nm LEDs, 600 nm actinic pump with 650 nm longpass-filtered tungsten lamp, and 690 nm actinic pump with 600 nm LEDs, respectively, up to 900 ps after electronic excitation. Strong scattering from the pump beam (specific location marked by the asterisk in each panel) was largely removed by subtracting the −2 ps TA trace from the subsequent traces, although residual scattering can be clearly seen in black (panels **B** and **C**). A weak positive band (light red color) is marked by the dashed rectangle at late times in panel (**C**). Key probe regions for dynamic analysis of the TA marker bands are highlighted by the color-coded vertical lines in each panel (see the associated least-squares fits with retrieved time constants in Supplementary Figure S2).

The time-resolved electronic spectra of CBCRs in buffer solution using ultrafast laser pulses (Figure 2) track the cofactor molecular population as it evolves on the excited-state potential energy surface (PES). There are four characteristic features in the fs-TA spectra: excited-state absorption (ESA), hot ground-state absorption (HGSA), ground-state bleaching (GSB), and stimulated emission (SE). Both ESA and HGSA bands are positive whereas GSB and SE bands are negative. The GSB feature overlaps with the ground state absorption band (see Figure 1D–F) which distinguishes it from the typically red-shifted SE and HGSA features. Notably, the measured TA signal intensity for Bpcb photoswitching processes (ca. ±18 mOD, OD is optical density, Figure 2A,C) is similar to that for Apcb at a similar sample concentration (OD ≈ 0.8 per mm at the P_r_ absorption peak) [15], whereas the less concentrated Bbv (OD ≈ 0.4 per mm at the P_fr_ absorption peak; see Table 1 in Section 2.1 above) displays much stronger TA intensity (~2–2.5 fold, Figure 2B,D). This result implies a larger excited-state transition oscillator strength [21,22] of the more conjugated BV cofactor than PCB cofactor in the CBCR pocket (see Figure 1) [18].

For corroboration, the raw experimental time-stacked fs-TA spectra (Supplementary Figure S1) exhibit the ultrafast dynamics of key electronic features, and the probe-dependent signal intensity (±5 nm of peak wavelength, see Supplementary Figure S2) least-squares fits yield multiple exponentials with characteristic time constants and amplitude weights [23]. Since the protein cofactor photoswitching process typically involves excited state intermediates undergoing parallel and/or sequential transitions, the observed broad spectral features could be highly overlapped so the retrieved time constants from the probe-dependent fits may be convoluted or combined into an averaged value. Therefore, it is imperative to analyze and compare the least-squares fits with the associated time constants and amplitude weights for multiple TA marker bands to identify common features for the excited-state chromophores (see each panel in Supplementary Figure S2 for a specific photoswitching process in various CBCR samples under study).

**Figure 3:**
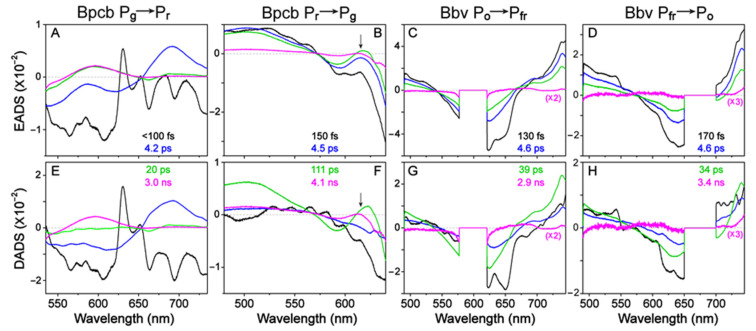
Global analysis of the fs-TA spectra during CBCR photoswitching processes. The underlying EADS/DADS of Bpcb P_g_ → P_r_, Bpcb P_r_ → P_g_, Bbv P_o_ → P_fr_, and Bbv P_fr_ → P_o_ transitions are shown in panels (**A**,**E**), (**B**,**F**), (**C**,**G**), and (**D**,**H**), respectively. The retrieved spectra are ordered from fastest to slowest lifetimes in black, blue, green to magenta traces, with the associated lifetimes color-coded and listed in insets. To remove pump scattering effect on data analysis, the probe window was truncated when the scattering signal was on the edge of detection window (panels (**A**,**B**,**E**,**F**)) or scattering region was set to zero (panels (**C**,**D**,**G**,**H**)). Black arrows in panels (**B**,**F**) show a weak ESA band at ~615 nm.

To help deconvolute the underlying cofactor species with pertinent time constants, global analysis was implemented to analyze TA data within the entire detection spectral and time window, allowing a holistic representation of the excited state electronic dynamics. The global analysis method typically employs two different models, evolution-associated difference spectrum (EADS) and decay-associated difference spectrum (DADS) [24]. In short, EADS uses multiple sequential states to model the spectra whereas DADS uses a parallel model with each species decaying with distinct lifetimes, which could provide complementary information for the complex TA data with overlapping spectral bands. The −2 ps (time delay, meaning that the probe pulse precedes the actinic pump pulse by 2 ps, hence serving as a useful background) TA spectrum was subtracted from the subsequent data traces to remove the strong scattering signal (some residuals can still be seen in Figure 2). During global analysis in Glotaran [25], the pump scattering was either set to zero if the feature was in the middle of the window or the window was trimmed when the scattering was at the edge so it would not interfere with the least-squares fits of the chromophore spectral features (Figure 3). Notably, the retrieved components from global analysis do not necessarily correspond to distinct molecular species, but rather, they represent a mathematical description of the spectral data with characteristic separable components and time constants in capturing the chromophore electronic dynamics.

The fs-TA spectral 2D-contour plot (Figure 2A) and global analysis results (Figure 3A,E) of Bpcb P_g_ → P_r_ photoconversion exhibit three major features that appear at the photoexcitation time zero: ~542 nm GSB (matching the steady-state absorption peak of P_g_ state in Figure 1E), 620 nm SE, and 690 nm ESA. These features correspond to the P_g_* (the asterisk denotes excited state) population. There is also rise of a positive feature around 595 nm that persists beyond the typical excited-state lifetime, hence it was assigned as an HGSA band of Lumi-G [15]. The 542 nm GSB band shows recovery dynamics of ~350 fs (10%), 3.5 ps (45%), 31 ps (29%), and 1 µs (16%) (Supplementary Figure S2A, black trace). The representative 1 µs component was used to model the long-lived population that does not return to original ground state within our detection time window. The most pronounced 690 nm ESA band shows clear decay dynamics with ~400 fs (9%), 4 ps (68%), and 27 ps (23%) time constants (Supplementary Figure S2A, red trace), which is accompanied by a clear rise of the 595 nm HGSA that has been attributed to the Lumi-G species of PCB cofactor in the “hot” electronic ground state [15]. Due to spectral overlap in the visible spectral region, the probe-dependent fit of the 595 nm band yields a ~200 fs (25%) and 3.4 ps (37%) decay of the initial negative feature (Supplementary Figure S2A, green trace, reminiscent of the adjacent GSB band dynamics) and a 21 ps (11%) rise of the Lumi-G HGS species that becomes apparent after ~4 ps (see the summary PES scheme below). The ~1 µs (27%) decay component was necessary for the fit since the long-lived signal eventually diminishes well beyond our current detection time window (see Methods in Section 3.3 below; a good separation of time constants allows the retrieval of longer time constants) [15].

Global analysis of the TA spectra for the photoinduced Bpcb P_g_ → P_r_ conversion requires five components to achieve a satisfactory fit, wherein the initial component fits a large coherent artifact with <5 fs lifetime (much lower than our cross-correlation time of ~120 fs [15,26]) so it was removed from the figure to focus on major electronic dynamics of the protein cofactor (see Figure 3A,E). The retrieved lifetimes were <100 fs, 4.2 ps, 20 ps, and 3 ns that largely match the probe-dependent fits (Supplementary Figure S2A). The sub-ps time constant is known to be Franck-Condon (FC) relaxation as the photoexcited wavepacket moves across the PES after electronic excitation [15,19,27,28]. This ultrafast step can be seen as clear and distinct features emerge in EADS (Figure 3A) and DADS (Figure 3E, black to blue trace). The 4.2 ps component likely tracks a partial twisting motion of the D ring in P_g_* as it moves toward a CI, consistent with literature and the well-accepted D-ring twist along the photoswitching coordinate [15,29]. This interpretation is also supported by the loss of P_g_* ESA and SE bands at ~690 and 620 nm (Figure 3A,E, blue to green trace), and the associated large amplitude weight that suggests a dominant relaxation pathway to the ground state. After the CI passage, the population diverges into two pathways. First, the twisted P_g_* can continue to twist and reach the Lumi-G state as the primary photoproduct species, which is evident by the ~21 ps rise of the Lumi-G HGSA band (Figure 3A,E, green to magenta trace; Supplementary Figure S2A, green trace). Second, the twisted P_g_* can reverse-twist back to P_g_ in the ground state as indicated by the ~31 ps GSB band recovery (Supplementary Figure S2A, black trace). The ~3 ns component from global analysis and ~1 µs component from the probe-dependent fits are representative of a weak fluorescence process and further protein-pocket-facilitated cofactor motions as Lumi-G fully converts to P_r_ in the electronic ground state [15,30].

The Bpcb P_r_ → P_g_ TA spectra (Figure 2C) and global analysis (Figure 3B,F) show clear P_r_* modes with an ESA band around ~520 nm and two GSB bands at 594 and 650 nm, with the latter two bands matching the main ground state absorption peak and its bluer vibronic shoulder (Figure 1E, red trace). The strong scattering from 650 nm actinic pump made the probe-dependent fits in that region unreliable (see black shade in Figure 2 and orange asterisk in Supplementary Figure S1C). We thus fit the cleaner 594 nm GSB band recovery dynamics with ~150 fs (34%), 4.7 ps (20%), 118 ps (30%), and 1 ns (16%) time constants (Supplementary Figure S2C, black trace). Meanwhile, the 503 nm ESA band exhibits similar intensity decay time constants of ~4.8 ps (13%), 254 ps (57%), and 2.5 ns (6%), but also requires a 28 ps (24%) component for a satisfactory fit (Supplementary Figure S2C, blue trace). The retrieval of two intermediate time constants (28 and 254 ps) instead of one 118 ps time constant (from GSB recovery) or 111 ps (from global analysis of the spectra within the detection window, see below) could be due to more complex dynamics when higher-lying electronic excited states are involved (e.g., the 503 nm ESA band due to the S_1_ → S_n_ transition) [20,31], although the general time scale of ~110–120 ps is consistent for the PCB cofactor excited-state relaxation along the S_1_ PES.

Notably, there is a discernible rise and decay of a positive band around ~615 nm (see Figure 2C, dashed rectangle box) which resembles the fs-TA data of Apcb [15] and could be attributed to a slightly twisted excited-state intermediate (P_r_*’). To reduce the pump scattering effect and confirm the delayed onset of this specific ESA band, we performed a control experiment using a dilute Bpcb sample (OD ≈ 0.2/mm at the P_r_ state absorption peak, about a quarter of the Bpcb sample concentration listed in Table 1 and presented in Figure 2C) and observed clear dynamics of a ~618 nm band with a much reduced scattering signal to the red side (Supplementary Figure S3). The 743 nm SE band shows similar decay dynamics to the bluer GSB band at 594 nm (Supplementary Figure S2C). The overall cofactor excited-state decay dynamics of Bpcb are faster than Apcb (see Section 2.5 below for a detailed comparison), suggesting that smaller residues in the chromophore vicinity of AnPixJg2_BV4 mutant [18] allow for faster ring-twisting events (likely accompanied by local environment relaxation) of the PCB cofactor [32].

Global analysis of the Bpcb P_r_ → P_g_ TA spectra yields ~150 fs, 4.5 ps, 111 ps, and 4.1 ns lifetimes. As discussed above, the 150 fs component reflects FC dynamics of the P_r_* species while the 4.5 ps component tracks the P_r_* → P_r_*’ transition. This key assignment was made previously in the Apcb P_r_ → P_g_ conversion by a pronounced rise and decay of the 616 nm ESA band of P_r_*’ species [15]. However, the more significant overlap with a stronger GSB band (versus that in Apcb) makes it more difficult to reliably fit the weak 615 nm P_r_*’ ESA band dynamics of Bpcb. Nevertheless, transient spectral signatures of the P_r_*’ state is evident from global analysis (Figure 3B,F, blue to green trace, see the black arrow) and the pertinent ~620 nm ESA band decays away on the 111 ps time scale, which likely corresponds to the non-equilibrium molecular movement toward a peaked CI [27,33] that can repopulate the original ground state and convert to the Lumi-R state [15,29]. Notably, the CI is also evident from the dominant loss of excited-state features with the 111 ps lifetime (Figure 3B,F, green to magenta trace), while the longer-lived 4.1 ns component with an SE band redder than ~670 nm (Figure 2C, with a more pronounced SE peak at ~743 nm in the dilute CBCR sample in Supplementary Figure S3) is likely owing to the fluorescence transition from P_r_* [15,29]. Overall, the P_r_ → P_g_ conversion is less efficient than the P_g_ → P_r_ conversion since the majority of excited-state species return to the original ground state [15,29,34], in accord with the observed longer lifetimes and the lack of prominent new TA features emerging at late times after 650 nm photoexcitation (see Figure 2C and Figure 3B).

For comparison, the fs-TA spectra of the newly engineered Bbv exhibit similar features for both the P_o_ → P_fr_ and P_fr_ → P_o_ transitions (Figure 2B,D), which differ from the PCB cases (Figure 2A,C). Although the observed TA bands are shifted in wavelength, they both exhibit a blue ESA band at the edge of our spectral window (<500 nm), a GSB band in the middle region (around 633 and 639 nm for P_o_ → P_fr_ and P_fr_ → P_o_ transitions, respectively, see Supplementary Figure S1B,D), and a strong red ESA band (>740 nm). An expanded spectral window to the bluer or redder probe regions is required to determine the exact locations for the blue and red ESA peaks; however, the increased similarity between the TA signatures of BV’s P_fr_ and P_o_ states versus PCB’s P_r_ and P_g_ states indicates the major effect of the intrinsic cofactor electronic structure, which likely causes the largely unchanged TA bands that are consistent with much less energy separation between the two conformers in BV (624 and 699 nm in Figure 1F, ~1719 cm^−1^) versus PCB (542 and 650 nm in Figure 1E, ~3066 cm^−1^). These common TA features in Figure 2B,D mainly track the P_o_* and P_fr_* electronic dynamics because they appear at the photoexcitation time zero and decay away with no clear wavelength shifts. It is notable that the P_fr_ GSB band around 690 nm overlaps with the pump scattering that is largely removed for TA analysis (Supplementary Figure S1D). Interestingly, all the least-squares fits of excited state dynamics show similar time constants for both conversions of the BV cofactor (Supplementary Figure S2B,D), which differs significantly from the PCB cofactor counterparts (Supplementary Figure S2A,C). For the P_o_ → P_fr_ conversion, the probe-dependent fits expose ~125 fs (43%), 4.6 ps (18%), and 32 ps (39%) time constants for the 499 nm ESA band, ~150 fs (48%), 4.6 ps (17%), 39 ps (34%), and 2.9 ns (1%) time constants for the 633 nm GSB band, and ~125 fs (27%), 4.6 ps (23%), 39 ps (49%), and 2.9 ns (1%) time constants for the 740 nm ESA band (see Supplementary Figure S2B). Similar dynamic components are apparent for these TA bands that report on the common excited-state decay pathways of a presumably homogeneous P_o_* population [35,36,37] as discussed in detail below.

For the P_fr_ → P_o_ conversion, the corresponding probe-dependent fits expose ~125 fs (35%), 4.8 ps (27%), 39 ps (37%), and 3.4 ns (1%) time constants for the 499 nm ESA band, ~129 fs (50%), 4.6 ps (21%), 37 ps (28%), and 3.4 ns (1%) for the 639 nm GSB band, and ~130 fs (31%), 4.9 ps (31%), 36 ps (34%), and 3 ns (4%) time constants for the 740 nm ESA band (see Supplementary Figure S2D), which reflect the common excited-state decay pathways of a presumably homogeneous P_fr_* population. Note that such a systematic analysis of independent measurements on reversible photoswitching processes of the same CBCR sample (e.g., Bbv with specific light irradiation conditions listed in Table 1 in Section 2.1 above) substantiates the robustness of the aforementioned time constants and amplitude weights for the underlying dynamic components retrieved after an actinic pump. As further corroboration, global analysis of the P_o_ → P_fr_ (Figure 3C,G) and P_fr_ → P_o_ (Figure 3D,H) transitions also output similar time constants of ~130 and 170 fs, 4.6 and 4.6 ps, 39 and 34 ps, and 2.9 and 3.4 ns, respectively. Similar to the Bpcb P_g_ → P_r_ conversion case (Figure 3A,E), the fit for a large coherent artifact was removed in the reversible photoswitching between P_o_ and P_fr_ states of Bbv to retrieve the intrinsic underlying time constants along the excited-state PES of the protein cofactor. Moreover, though the EADS show the blue trace (4.6 ps lifetime) decaying into green trace (~35 ps lifetime, Figure 3C,D), the corresponding DADS show the green trace intensity higher than blue trace in both ESA regions (below 550 nm and above 720 nm, Figure 3G,H). This finding is indicative of some deviations from a simple kinetic model that the 4.6 ps component precedes the 35 ps component (see below).

To confirm these key observations and results for the Bbv photoswitching mechanisms, we performed the following control experiments: (1) Fs-TA data sets were collected at least twice with the freshly prepared protein samples on different days in the laser lab to ensure reproducibility. (2) The steady-state absorption spectra of the P_o_ and P_fr_ conformers were taken before and after every TA experiment to confirm that the protein samples maintain their photoswitching capabilities. (3) Control TA experiments using 600 nm actinic pump with 600 nm LEDs (Supplementary Figure S4A) and 704 nm actinic pump with the 650 nm longpass-filtered tungsten lamp (Supplementary Figure S4B) were conducted to ensure that the TA data in Figure 2B,D were not just collecting the same conformer or mixed species. (4) A visual confirmation was achieved that the actinic pump could convert the protein samples by holding the sample cuvette in the beam path before it was focused [15]. (5) A redder actinic pump at 704 nm was used and similar TA features were observed for the P_fr_ → P_o_ transition (see Supplementary Figure S5 and Figure 2D). With all these strategic, useful, and interwoven control experiments substantiating the aforementioned results, the observed similar dynamics of the P_o_ → P_fr_ and P_fr_ → P_o_ transitions infer a very alike or even “purely” reversible excited-state pathway of the BV cofactor in AnPixJg2_BV4.

The major differences between the aforementioned reversible photoswitching dynamics are the amplitude weights of the two intermediate components retrieved on the ps time scale. The P_o_ → P_fr_ conversion (Supplementary Figure S2B) shows about half the weight for the ~5 ps component (18%, 17%, and 23%) compared to the ~35 ps component (39%, 34%, and 49%) for the blue ESA, GSB, and red ESA bands, respectively. In contrast, for the corresponding TA marker bands, the P_fr_ → P_o_ conversion (Supplementary Figure S2D) shows approximately equal weights for the ~5 ps (27%, 21%, and 31%) and ~35 ps (37%, 28%, and 34%) components. This experimental finding poses two interesting questions. (1) Why does Bbv exhibit similar photoswitching dynamics in both directions whereas the Apcb and Bpcb P_g_ → P_r_ and P_r_ → P_g_ conversions show different excited-state pathways? (2) Why are the amplitude weights of two largely conserved intermediate ps time constants the major differentiating factor between the Bbv P_o_ → P_fr_ and P_fr_ → P_o_ photoconversion?

### 2.3. Altered Excited-State Dynamics Arise from an Interplay between PCB or BV Cofactor and AnPixJg2 or AnPixJg2_BV4 on Ultrafast Time Scales

To answer these fundamental questions, we first examined the differences between PCB and BV cofactors in the AnPixJg2_BV4 pocket. The location-shifted BV cofactor in the BV4 pocket moves the A ring closer to its H-bonding partners Asp291 and Trp289 [18]. The distance between the cofactor A ring nitrogen atom and the Asp291 oxygen atom decreases from ~1.95 Å in the Apcb P_r_ state crystal structure (PDB ID: 3W2Z) [8] to 1.76 Å in the Bbv P_fr_ state crystal structure (PDB ID: 5ZOH at 1.60 Å resolution) [18]. The distance between the cofactor A ring carbonyl oxygen and the Trp289 nitrogen is ~0.21 Å closer in Bbv (2.76 Å) than Apcb (2.97 Å). The closer H-bonding partners and the extra electron density in the A ring would effectively restrict it from large twisting motions, while a slight twist of the A ring has been reported as a component in the photoconversion process [15,37,38]. By better stabilizing the A ring inside the protein pocket, more of the photoconverting energy could be localized at the D ring with a reversible clockwise/counterclockwise twist (i.e., along the same isomerization pathway but in opposite directions) by reducing motions away from the D ring.

As the D ring twists, there are two dihedral angles that correlate with the conversion between P_fr_ and P_o_ conformers. The dihedral closer to the D ring (D-dihedral) likely causes a smaller twisting motion than the dihedral closer to the C ring (C-dihedral, see Figure 1A–C), since the D-dihedral twist is a volume-conserving motion whereas the C-dihedral twist likely increases the volume [39,40,41]. Upon excitation of P_fr_ to P_fr_*, the conjugated double bonds acquire more single-bond character and vice versa [33,42,43], in accord with previous studies showing that the C15=C16 bond (directly connecting to the D ring) is responsible for the major twisting between the 15*Z*/*E* conformers [44,45,46]. By analyzing both dihedral angles for the available Apcb P_r_ state and Bbv P_fr_ state crystal structures, the C-dihedral (CCCC) around the bridge single bond (36.08° vs. 51.12°) is more twisted in Bbv whereas the D-dihedral (CCCN) around the bridge double bond (26.96° vs. 0.83°) is more twisted in Apcb from the pyrrole ring plane [8,15,18]. The BV4 mutations, specifically the more critical F308T and I336V, help to accommodate the shifted cofactor around the D ring due to less bulky residues [18]. Notably, if we attribute the observed 4.6 and 34 ps in the Bbv P_fr_ → P_o_ photoconversion (Figure 3D) to the small-scale D-dihedral and large-scale C-dihedral twisting motions, respectively, we could then expect a smaller and larger time constant from the cofactor TA dynamics in Apcb due to the aforementioned starting geometry. Indeed for the C-dihedral twist, we observed a 217 ps component for the Apcb P_r_ → P_g_ transition [15], and a 111 ps component for the Bpcb P_r_ → P_g_ transition (Figure 3B) likely owing to an intermediate geometry of the D ring in between the Apcb P_r_ and Bbv P_fr_ conformers. In contrast, for the D-dihedral twist on the few ps time scale, it is less sensitive to the initial geometry alone mainly due to its close interaction with an adjacent Val336 or Ile336 (in Bbv, Bpcb or Apcb, see Figure 1A–C) that could affect the faster time constant (in preparation for the subsequent larger twist motion) [20,47].

It is likely that Bpcb also takes advantage of the extra space around the cofactor and adopts a slightly shifted orientation from Bbv. The larger area around the D ring also explains the overall faster photoswitching dynamics in Bpcb than Apcb [32]. The slightly slower ~4 ps component in Bpcb P_g_ → P_r_ than ~3 ps in Apcb P_g_ → P_r_ conversion [15] can be explained by the shifted PCB cofactor in the AnPixJg2_BV4 pocket which would bring the D ring closer to Val336 and somewhat hinder the initial smaller twist (i.e., D-dihedral). Although the smaller twisting motion becomes slower, the larger twist (i.e., C-dihedral) occurs faster in the P_g_ → P_r_ photoconversion of Bpcb (~20 ps) than Apcb (~30 ps), both retrieved from global analysis of the corresponding TA spectra after 525 nm photoexcitation. We note that the larger twist occurs in the electronic ground state past the CI, likely owing to the metastable P_g_* species that undergoes a more downhill and efficient process than the more “trapped” P_r_* species that requires both twisting processes in the electronic excited state [15,29]. Interestingly for the latter case, the P_r_ → P_g_ photoconversion of Bpcb (~4.5 and 111 ps) also occurs faster than Apcb (~13 and 217 ps) involving presumably small and large ring-twisting motions of the protein cofactor, both retrieved from global analysis of the corresponding TA spectra after 650 nm photoexcitation [15].

For Bbv, because the photoswitching pathways are fully reversible with largely conserved time constants, the amplitude weights from the TA signal intensity fits become crucial in gaining mechanistic insights into the excited-state pathways. Since Bpcb does not exhibit such a reversible TA pattern during photoswitching, the observed “fully reversible” pathway seems to be unique to Bbv (i.e., both AnPixJg2_BV4 pocket and BV cofactor are needed). The probe-dependent least-squares fits implement a parallel model of multiple processes with a common time zero, although it does not mean that the fastest component occurs first. By inspecting the Apcb and Bpcb photoswitching scheme, the 3–4 and 20–30 ps components likely represent the aforementioned small and large twisting processes, respectively, although the detailed assignments to excited- or ground-state dynamics need to come from the TA spectral analysis. The amplitude weight of each time constant is representative of how dominant the associated pathway is for energy dissipation. Since the Bbv TA bands emerge around photoexcitation time zero and then decay, while the peak wavelength remains largely unshifted up to 900 ps (Figure 2B,D), the observed TA band intensity dynamics likely track the excited-state population change along the ring-twisting coordinates. Notably, a larger twist could cause a larger change of the electronic transition oscillator strength for the resultant conformation than that of a smaller twist, which may contribute to the apparent TA band intensity decay dynamics. We thus focus on the change of amplitude weight ratios instead of exact weights for ensuing analysis.

In the P_fr_ → P_o_ conversion of Bbv, the equal weights of the ~5 ps and 35 ps components (Supplementary Figure S2D) suggest that a 5 ps smaller twisting motion would populate the P_fr_*’ state before undergoing the 35 ps larger twisting motion en route to the CI. The less energy-consuming and more facile 5 ps twist could reduce the chances of other nonradiative pathways that compete with the 35 ps twist, while the initial small twist out of the more stabilized P_fr_ state may be required to help the D ring move away from the adjacent Val336 to allow more room for the subsequent large twist in the excited state (Figure 1C). In the P_o_ → P_fr_ conversion of Bbv, an unexpected observation that the ~35 ps larger twist being twice as dominant as the 5 ps smaller twist implies that the larger twisting motion occurs first. The more energy-consuming and less facile 35 ps twist would bring the excited state closer to the ground state, allowing for more nonradiative processes to compete with the smaller twist. This would effectively cause the weight of the smaller twisting component to drop in comparison with the larger twist [20,21]. After the smaller twist, the remaining population could pass through a CI and continue to undergo cofactor ring twist and the surrounding protein pocket relaxation to reach the isomerized state (see Section 2.5 below and Graphical Abstract for illustration). We note that if we attributed the ~5 and 35 ps to inhomogeneous species of P_o_* undergoing excited-state energy relaxation in parallel, then it would be presumptuous to explain the same time constants from two inhomogeneous subpopulations of P_fr_* because the two conformer states have different cofactor conformations in their respective surrounding protein pockets upon electronic excitation [8,38].

In both Bbv conversions, most of the population returns to the original ground state. The lack of a long-lived GSB recovery is evident (Supplementary Figure S2B,D) that per excitation pulse, the TA signal from any photoconverted population is likely within our experimental signal-to-noise ratio. Although having an apparently lower photoconversion efficiency than Apcb, Bbv still exhibit a fast photoconversion and can operate effectively under cw or ambient light sources for potential optogenetic applications [3,7,17].

### 2.4. Ground State Femtosecond Stimulated Raman Spectroscopy Sheds Light on Structural Factors That Rationalize the Unique Reversible Photoswitching Pathways in Bbv

**Figure 4:**
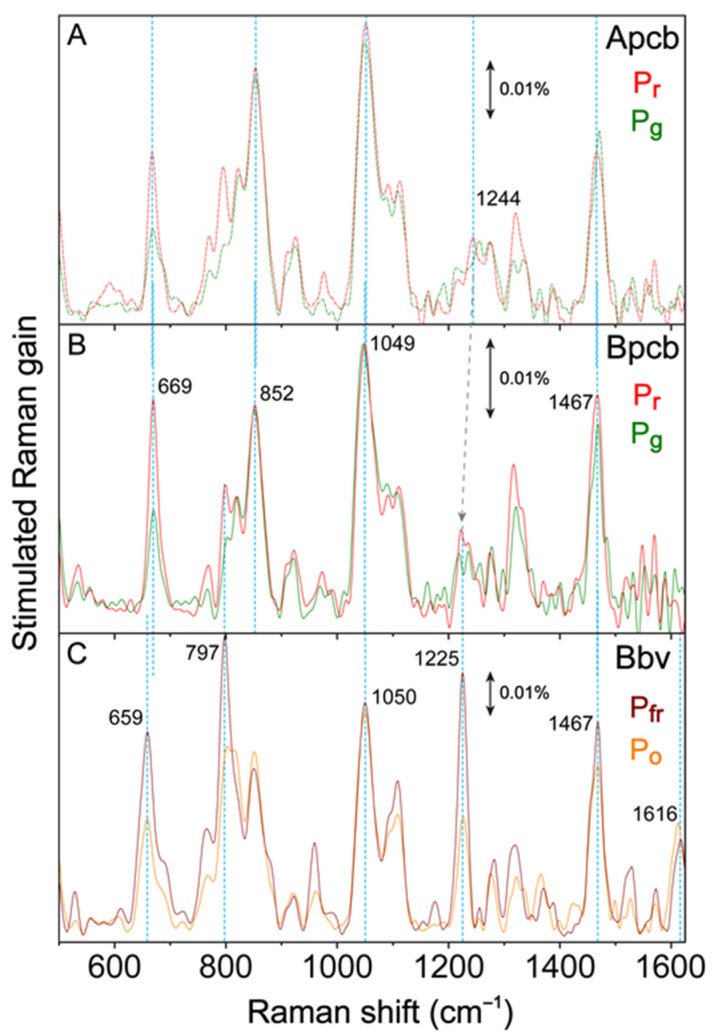
Raman signatures of various cofactors in related CBCR conformers. GS-FSRS spectra of (**A**) Apcb and (**B**) Bpcb P_r_ (red) and P_g_ (green) species using 505 and 650 nm LEDs, respectively, as well as (**C**) Bbv P_fr_ (dark red) and P_o_ (orange) species using 600 nm LEDs and a 650 nm longpass-filtered tungsten lamp. The ps 803 nm Raman pump was used to minimize the pump-induced conversion of the cofactor species, with the fs Raman probe set at the Stokes side. Vertical dotted lines and a dashed arrow denote the peak frequency shifts between key vibrational modes of related CBCR cofactors. The double-headed arrows denote the stimulated Raman gain magnitude of 0.01% in each panel.

To help elucidate the atomic structures of Bpcb and Bbv conformers, GS-FSRS experiments were performed to obtain the vibrational signatures of Apcb, Bpcb, and Bbv (Figure 4). Respective LEDs were implemented to collect the Apcb P_r_ and P_g_ (Figure 4A), Bpcb P_r_ and P_g_ (Figure 4B), as well as Bbv P_fr_ and P_o_ conformers (Figure 4C). With the pre-resonance ps 803 nm Raman pump, the fs broadband Raman probe on the Stokes side was used to effectively prevent notable dispersive line shapes of the cofactor chromophore [20,48]. The collected spectra in Figure 4A match our previous experimental results on Apcb using various Raman pump wavelengths at 792, 678, and 596 nm with Raman probe on the Stokes or anti-Stokes sides [15]. As a control experiment, we also collected the anti-Stokes FSRS spectra with a bluer Raman probe than the Raman pump (Supplementary Figure S6) [22,48,49], which exhibit much stronger peaks due to the resonance enhancement achieved by the Raman probe with respect to the ground state absorption peaks of P_fr_ (699 nm) and P_o_ (624 nm), further confirming the vibrational peak frequencies in S_0_ [28,48,50,51]. Using the Gaussian software [52], density functional theory (DFT) calculations at B3LYP level with 6–31G(d,p) basis sets were performed on the BV cofactor to visualize the vibrational normal modes (see Experimental Methods in Section 3.4 below). A frequency scaling factor 0.97 was applied to match the DFT-calculated spectrum to the GS-FSRS spectrum of the Bbv P_fr_ conformer (Supplementary Figure S7). The discrepancy in the Raman peak intensities is likely due to the DFT calculations only treating the cofactor quantum mechanically in vacuo without the protein pocket. More accurate hybrid quantum mechanics/molecular mechanics (QM/MM) methods would help model the CBCR protein with the embedded cofactor as a whole [37,45,53,54] which could complement our experimental capabilities in ultrafast laser spectroscopy. The description of major vibrational modes of the P_fr_ state can be seen in Supplementary Table S1.

Overall, Apcb, Bpcb, and Bbv share marker bands common to bilin photosensors at ~1051 and 1466 cm^−1^ [35,55]. PCB and BV cofactors exhibit similar vibrational motions with small frequency shifts (Figure 4). The 1051 cm^−1^ mode is localized to the D-ring deformation with strong C–H sidechain wagging. The 1466 cm^−1^ is attributed to the B and C ring-deformation and CH_3_ rocking. Both of these modes are stabilized by the conserved His322, Trp289, and Tyr352 in the CBCR pocket [18]. The overall higher intensity of the red-absorbing species (P_r_ in Apcb and Bpcb, and P_fr_ in Bbv) is attributed to better resonance enhancement with the 803 nm Raman pump. Moreover, the similar peak intensity at ~1050 cm^−1^ for P_o_ and P_fr_ of Bbv while most other modes display reduced intensities in P_o_ can be rationalized by the vibronic coupling matrix that yields the mode-dependent change of resonance conditions [15,20], which indicates the importance of light-induced D-ring motions that connect these conformers. Key differences between the vibrational spectra of PCB (Figure 4A,B) and BV (Figure 4C) can be seen by the red-shifted modes around 660 and 800 cm^−1^, and in the 1200–1400 cm^−1^ region.

The ~660 cm^−1^ peak region reveals key differences in electronic conjugation between the PCB and BV cofactors. This Raman mode involves collective C–C and C–H wagging motions in the conjugated cofactor ring system. A frequency redshift of 10 cm^−1^ from the ~669 cm^−1^ mode in Apcb and Bpcb (Figure 4A,B) to 659 cm^−1^ mode in Bbv (Figure 4C) is rationalizable by two extra double bounds in the A and D rings of Bbv cofactor (see Figure 1C). In Bbv, the peak doublet around 825 cm^−1^ shows a clear blueshift of the lower-frequency mode from P_fr_ (797 cm^−1^) to P_o_ (809 cm^−1^). Since the associated vibrational motions involve N–H wagging from the A, B, and C rings with C–H wagging on the CD bridge [15,55], the frequency blueshift is likely due to a twisted D ring that disrupts the H-bonding of Trp289 to A ring. In Apcb and Bpcb, the lack of a strong peak around 800 cm^−1^ could be attributed to a different H-bonding network of the A ring with reduced electric polarizability (one less double bond). In the 1200–1400 cm^−1^ region, Bbv exhibits a prominent 1225 cm^−1^ mode. In Bpcb and Apcb, this mode is weak but displays a noticeable redshift from Apcb to Bpcb (~1244 to 1225 cm^−1^, see the tilted arrow from Figure 4A,B). In both PCB and BV calculations [15], since this mode involves delocalized N–H rocking and AB bridge C–H rocking motions, the clear peak enhancement in Bbv (Figure 4C) further corroborates the shifted, stronger H-bonded A ring in the more conjugated BV cofactor. The redshift in Bpcb compared to Apcb is also consistent with the BV4 mutations that allow the PCB cofactor to adopt a slightly shifted position inside the protein pocket and establish better H-bonding interactions, yet the largely unchanged Raman peak intensity around 1235 cm^−1^ indicates that the mode-dependent electric polarizability is dictated by the intrinsic chromophore electronic structure (see the clear peak intensity ratio and line width change from Figure 4A,B to Figure 4C) [20,29].

### 2.5. Contrasting Excited-State Potential Energy Surfaces for Reversible Photoswitching of PCB and BV Cofactors in the AnPixJg2_BV4 Pocket

**Figure 5:**
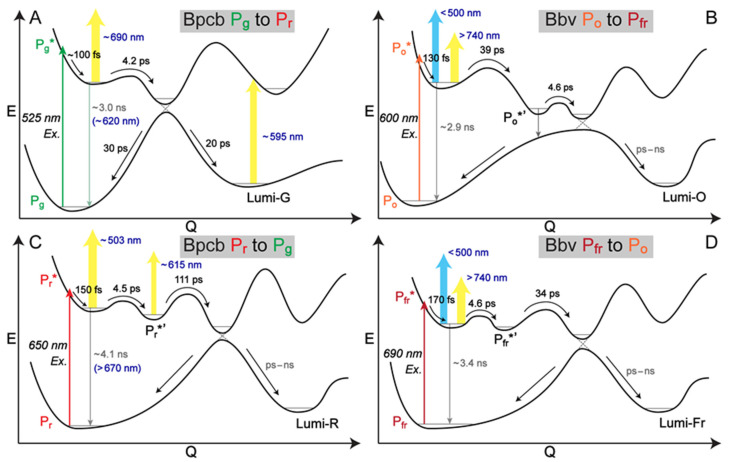
Representative potential energy surfaces of Bpcb (**A**) P_g_ → P_r_, (**C**) P_r_ → P_g_, and Bbv (**B**) P_o_ → P_fr_, (**D**) P_fr_ → P_o_ photoconversion. The photoexcitation (Ex.), ESA, SE, GSB, and HGSA transitions are illustrated by the color-coded vertical arrows. The arrow lengths are not drawn to scale with the exact energy gaps. Reaction scheme is denoted by curved arrows with characteristic time constants retrieved from global analysis of the corresponding TA spectra in Figure 2 and Figure 3. A minor fluorescence component is shown by a dim downward arrow. The ps-ns time scales for Lumi state formation in panels (**B**–**D**) indicate a long-lived state beyond our current detection time window. The horizontal axis “Q” represents the dominant cofactor ring-twisting motions that could change as time progresses after photoexcitation, while the protein environment continuously contributes to the cofactor nuclear motions.

With all the correlated electronic and vibrational signatures from steady-state to time-resolved regime [15,20], we could sketch the overall reversible photoswitching pathways of Bpcb and Bbv (Figure 5). Bpcb adopts the similar reaction scheme as Apcb as the BV4 mutations do not affect the major pathways of the embedded cofactor. The less bulky residues (particularly Thr308 and Val336, Figure 1B) in Bpcb allow the D ring more room to twist, decreasing the excited-state dynamics time constants [15,36]. In the Bpcb P_g_ → P_r_ conversion (Figure 5A), the P_g_* species undergoes FC relaxation on the ~100 fs time scale, then approaches a CI on the ~4.2 ps time scale. The slightly shifted cofactor in Bpcb makes the initial twist slightly slower than that in Apcb (~2.7 ps) [15]. After the CI, the cofactor population likely continues twisting to the converted Lumi-G state or twisting back to P_g_ on the 20–30 ps time scales. This step is evident by a ~20 ps component in the Lumi-G rise (HGSA at ~595 nm in Figure 2A) and a ~30 ps component in the GSB recovery (Supplementary Figure S2A). As for the opposite P_r_ → P_g_ conversion (Figure 5C), after FC relaxation the P_r_* species transitions into a twisted P_r_*’ intermediate with a 4.5 ps time constant, which requires further ring twists into a CI on the 111 ps time scale. The P_r_*’ state can be seen by the transient rise and decay of a weak ESA band around 615 nm (Figure 2C and Supplementary Figure S3), corroborated by a stronger ESA band around 616 nm with similar dynamics for the same PCB cofactor inside the AnPixJg2 pocket (i.e., Apcb) [15].

On the long time scale, since the 3–4 ns minor component from global analysis of Bpcb (Figure 3A,B,E,F) is an average value of all the long processes going beyond our current time window of ~1 ns, the pertinent contributors could be fluorescence from P_g_* species (see the 620 nm SE band in Figure 2A and PES in Figure 5A) or P_r_* species (see the >670 nm SE band in Figure 2C and PES in Figure 5C), and the long-lasting photoproduct state that does not return to the original ground state on this time scale (e.g., Lumi-G in Figure 5A and Lumi-R in Figure 5C). In analogy, the similar ~3 ns minor component from global analysis of Bbv (Figure 3C,D,G,H) could involve weak fluorescence from P_o_* and P_fr_* species plus the long-lasting photoproduct states Lumi-O and Lumi-Fr in Figure 5B and 5D, respectively. It is interesting to note that these minor components with 1–4% amplitude weights were consistently retrieved from both the red ESA band (~740 nm) and GSB band (633–639 nm) of Bbv (Supplementary Figure S2B,D), indicating that a small percentage of the excited-state population goes from the excited state to the original ground state on the ~3 ns time scale (a typical radiative transition lifetime), which could be due to the aforementioned weak fluorescence component. The absence of a prominent SE band could be due to spectral overlap with the strong and broad ESA band on the red side (Figure 2B,D and Figure 3C,G,D,H), and any mismatch between the ESA and GSB dynamics could imply some deviations from a direct S_1_-to-S_0_ transition. These long-time-scale processes could benefit from future in-depth studies with other experimental and computational techniques (see below) that are beyond the scope of this work.

Notably, the engineered Bbv undergoes a more “purely” reversible photoswitching pathway as reflected by the highly conserved excited-state time constants from either conformer of the cofactor. The P_fr_ → P_o_ conversion (Figure 5D) adopts a similar scheme to the P_r_ → P_g_ conversion of the PCB samples from the thermally equilibrated state to the metastable state. The similarly weighted ~4.6 and 34 ps time constants infer that the 4.6 ps small-scale twisting motion occurs prior to the 34 ps larger twist, which is characteristic of the D-dihedral change acting as a preparatory step [28,56] for the C-dihedral change (see details in Section 2.3 above). In contrast, starting from the metastable state with a more downhill relaxation, the higher-weighted ~39 ps larger twisting motion could generate a P_o_*’ species that is energetically closer to the ground state, which allows for other nonradiative processes (depicted by a vertically downward gray arrow in Figure 5B) to more effectively compete with the 4.6 ps small-scale twist leading into to a CI region before the Lumi-O formation. Given this kinetic scheme, we do not expect a significant accumulation of transient P_o_*’ species since the 4.6 ps outgoing rate is much faster than the 39 ps incoming rate, which likely explains the lack of clear TA features from the P_o_*’ species. As a result, the observed TA dynamics (Figure 2B) mainly reflect the ultrafast multi-exponential decay of P_o_* species (Figure 5B), consistent with kinetic models with reversible reactions connecting all the transient species. In particular, the DADS feature with a 4.6 ps lifetime (Figure 3G) does not reflect the P_o_*’ species per se, instead, it mathematically tracks the 4.6 ps kinetic component (along the P_o_*’-to-CI pathway) with a similar spectral profile as the initial ~130 fs and subsequent 39 ps components (i.e., P_o_* species) as shown in the EADS analysis (Figure 3C).

Recent reports on the ultrafast dynamics of a red/far-red bathy bacteriophytochrome named PaBphP that binds BV inside the GAF domain (part of a classical PAS-GAF-PHY architecture) [32,36] provide a useful comparison. The forward ^15*Z*^P_r_ → ^15*E*^P_fr_ photoconversion utilizes a two-step process on the excited state with a 2–207 ps local environment (e.g., protein residue sidechains, internal/trapped water molecules [55,56,57], elaborate H-bonding networks) relaxation and a 375 ps isomerization to the CI [32]. For comparison, the reverse P_fr_ → P_r_ photoconversion (with higher bond strain/distortion to release) goes through an excited-state bifurcation on the much faster ~1 and 4 ps time scales where both wavepackets reach the CI [36]. The pertinent few-ps photoisomerization step around the C15=C16 bond that significantly shifts the cofactor D-ring with respect to a nearby protein pocket residue is consistent with our assignment of the 3–5 ps component to the D-dihedral/small-scale twisting motion (see Section 2.3 above). By evaluating the conformational differences between P_fr_ and P_r_ states of PaBphP via X-ray crystallography, the BV cofactor exhibits distinct conformations of the A, B, C, and D rings [58], which underlie the significant difference between forward and reverse pathways mediated by different residues in the extensively rearranged H-bonding network. In sharp contrast, stabilizing the A, B, and C rings through largely conserved H-bonding and π-π stacking (e.g., between Trp289 and D ring) in the BV4 pocket [18] could therefore allow a rather unique and “purely” reversible photoconversion pathway of the BV cofactor (between the P_o_ and P_fr_ states of Bbv, Figure 1F) as observed in this work (Figure 2 and Figure 3, Supplementary Figures S1 and S2). Notably, the focus here is the elucidation of reversible photoswitching between distinct (not necessarily pure) conformers in the protein pocket.

The more homogeneous, thermally equilibrated populations of Bpcb and Bbv could also be related to the protonation state of the adjacent His322 [37]. QM/MM calculations indicate that the His322 protonation state is a trigger for structural heterogeneity. In the crystal structure of the P_fr_ state of AnPixJg2_BV4 [18], His322 shares two H-bonding partners: one is the B ring propionate, the other is a local water molecule that also H-bonds to the D ring nitrogen site (–NH group). The His318Tyr mutation in BV4 removes the original H-bond between His318 and the B ring propionate in AnPixJg2 [8] and would thus increase the H-bonding interaction between B ring and His322 [18]. For the other H-bonding partner, since both PCB and BV cofactors undergo some position shift in the BV4 pocket, the D ring likely has more tendency to move away from His322. The resultant singly protonated state of His322 (as an H-bond donor to the B ring propionate) would support a more homogeneous cofactor population [37], which is evident from the “native” steady-state electronic absorption spectra that show Bbv (Figure 1F, black trace) being more homogeneous than Bpcb (Figure 1E, black trace). The homogeneous population also supports Bbv in adopting a sequential kinetic model instead of a parallel model to undergo excited-state electronic and structural relaxation of the BV cofactor with continuous active-site motions in S_1_ [30,32], followed by slower thermal relaxation events of the chromophore and protein matrix in the electronic ground state (S_0_). By exciting a mostly homogeneous ground state population, it is unlikely to have the transient wavepacket branch into different pathways with notably different time constants (e.g., ~5 and 35 ps) that both reach the CI.

Meanwhile, the P_o_ conformer of Bbv is likely more homogeneous due to its higher absorption peak than the P_g_ conformers of Apcb and Bpcb (Figure 1F orange trace vs. Figure 1D,E green traces, all relative to their corresponding redder-absorbing peaks), because a larger extinction coefficient could be indicative of a more stabilized P_o_ state of Bbv. For corroboration, implementing the BV4 mutation to AnPixJg4 (the fourth GAF domain of AnPixJ) leads to a rapid P_o_-to-P_fr_ dark reversion that is ~400-fold faster than that of AnPixJg2_BV4 (Bbv), while the relative P_o_/P_fr_ absorption peak intensity ratio (0.65, peak wavelengths at 628 nm/700 nm) [18] is indeed smaller in AnPixJg4_BV4 than that in Bbv (0.85, peak wavelengths at 624 nm/699 nm, Figure 1F). The BV-binding efficiency of AnPixJg4_BV4 (44%) is also significantly lower than AnPixJg2_BV4 (75%). Further investigation of AnPixJg4_BV4 using a similar combination of fs-TA and GS-FSRS to this work could help to unveil the mechanistic origin for the fourth GAF domain to exhibit greatly accelerated dark reversion on the ground state, shedding light on the effect of cofactor-protein-pocket interplay on the apparent TA features versus the thermally equilibrated P_fr_ state. Knowledge about the effects of specific attributes around the cofactor and how they contribute to the overall photoswitching dynamics would enable the protein engineers to effectively tailor these photoswitching proteins for specific applications.

To further decipher the multidimensional [20,56,57] photoswitching dynamics, we expect the improved molecular dynamics (MD) simulation methods with higher accuracy and longer duration [26,37,55,57,59] and other spectroscopic techniques to paint a holistic and accurate portrait as the molecular system is excited, photoconverting, and relaxing back to its thermally stable dark-adapted state [29,55]. Since the metastable light-adapted P_o_ and P_g_ crystal structures are challenging to obtain, QM/MM methodology has advanced in closely matching the calculated absorption spectra of protein conformers with their experimental counterparts, including recent successes on a similar red/green absorbing CBCR named Slr1393g3 with its P_r_ and P_g_ crystal structures both solved [38,54]. By implementing similar QM/MM calculations on Bbv, more accurate vibrational and structural insights into the P_o_ conformer could be gained to augment our current understanding of the P_fr_ conformer (Figure 4 and Table S1) with its available crystal structure [18]. Moreover, a systematic calculation of the excited-state free energies of the BV cofactor with various D-ring twisting geometries and intermediates (see Section 2.3 above) in the protein pocket can shed more light on the ultrafast reversible photoswitching pathway between the P_o_ and P_fr_ conformers of AnPixJg2_BV4.

In addition, an expanded spectroscopic toolset from ultrafast regime (typically fs-to-ns time scales as reported here) to the more macroscopic time scales (ns-to-ms or longer) could better identify long-lived or later intermediates (e.g., Lumi-G and Lumi-R in Apcb and Bpcb, and Lumi-O and Lumi-Fr in Bbv), which may dictate the forward/reverse reaction speed and yield [5,29,60,61]. These Lumi states are likely similar to the photoproduct species in our current experiments on the ns time scale (Figure 5), however, they require further protein relaxation and chromophore pocket rearrangement on the electronic ground state to eventually reach the final product state. For example, a TA setup with pulsed (100 ns) LED-based broadband probe has expanded the time window from ns to ms and identified two intermediates between Lumi-G_2_ and P_r_ after 532 nm excitation of P_g_ of NpR6012g4 [62], and recent applications in conjunction with fs-TA were able to elucidate a series of meta states that emerge after the Lumi state formation in AnPixJg2 as well as NpR6012g4 with a strong sequence homology (both are canonical red/green CBCRs) [61]. In aggregate, by combining multiple techniques such as TA, FSRS, QM/MM, X-ray crystallography, and NMR, the resultant comprehensive information can delineate the complex photoconversion of CBCRs and other photosensing proteins as they convert from one state to another on various time scales. Such a more complete understanding of the working mechanisms of these fascinating nanomachines can help more bioengineers to better predict, design, prepare, and tune the next generation of bioimaging and optogenetic toolsets to advance life sciences and improve human health.

## 3. Materials and Methods

### 3.1. Protein Expression

The His-tagged AnPixJg2_BV4 inserted into pET28a vector (Novagen, Sigma Aldrich, St. Louis, MO, USA) was constructed in our previous study [18]. The plasmid was transferred into the *E. coli* strain C41 (Cosmo Bio, Tokyo, Japan) harboring the PCB synthetic system (pKT271-C0185) or BV synthetic system (pKT270) for protein expression. The bacterial cells were grown on Lysogeny Broth (LB) agar medium at 37 °C and selected by kanamycin and chloramphenicol (each final concentration set at 20 μg/mL), then cultured in 1 L LB medium at 37 °C until the cell optical density (OD) at 600 nm reached a range of 0.4–0.8. Isopropyl β-D-1-thiogalactopyranoside (IPTG at final concentration of 0.1 mM) was then added into the culture media and these cells were cultured at 18 °C overnight to induce protein expression.

### 3.2. Protein Extraction and Purification

After protein expression was induced, the culture broth was centrifuged at 5000 *g* for 15 min to collect cells and then frozen at −80 °C. The cells were suspended in a lysis buffer (20 mM HEPES–NaOH at pH = 7.5, 0.1 M NaCl, and 10% (*w/v*) glycerol) and disrupted by Emulsiflex C5 high-pressure homogenizer at 12,000 psi (Avestin Inc., Ottawa, On, Canada), The 4-(2-hydroxyethyl)piperazine-(2-ethanesulfonic acid) or HEPES aqueous buffer solution was used because it has higher stability in maintaining the physiological pH values of cell culture media despite changes in CO_2_ concentration. The mixtures were then centrifuged at 165,000× *g* for 30 min to remove pellets, and the collected solutions were filtered through a 0.2 μm membrane. After adding imidazole (30 mM final concentration), the solution was loaded onto a nickel-affinity His-trap column (GE Healthcare, Chicago, IL, USA). After washing with lysis buffer containing 30–100 mM imidazole, the His-tagged proteins were purified using lysis buffer containing 100–400 mM imidazole with a linear gradient system (1 mL/min) for 15 min. The purified proteins were incubated with 1 mM EDTA on ice for 1 h and then dialyzed against the lysis buffer to remove the residual imidazole and EDTA. This detailed process to make Bpcb (green/red) and Bbv (orange/far-red) samples in this work was identical to the one that we used to prepare Apcb (green/red) samples [15], and all the CBCR samples were prepared in the aforementioned HEPES buffer solution at physiological pH (7.5) and the specific concentrations (see Table 1 in Section 2.1 above) for all the steady-state and time-resolved spectroscopic measurements as reported herein.

### 3.3. Femtosecond Transient Absorption (fs-TA) and Ground-State Femtosecond Stimulated Raman Spectroscopy (GS-FSRS) with Various Light Irradiation Conditions

Our fs-TA and GS-FSRS setups have been built upon a Ti:sapphire-based laser oscillator (Mantis-5, Coherent, Inc., Santa Clara, CA, USA) and regenerative amplifier (Legend Elite-USP-1K-HE, Coherent, Inc.), which produce fundamental pulses of ~800 nm center wavelength, 3.7 W average power, and 35 fs duration at 1 kHz repetition rate [57,63]. Tunable fs photoexcitation pulses were generated through a home-built two-stage noncollinear optical parametric amplifier (NOPA) [64]. The desired output laser wavelength was selected in the first NOPA by overlapping a supercontinuum white light (SCWL) and a 400 nm pump, which were generated by focusing a portion of the fundamental pulses on a thin sapphire crystal and a BBO crystal, respectively. This output pulse was used as the seed and further amplified by the second NOPA with a stronger 400 nm pump. The probe pulse was also an SCWL but generated through a 2-mm-thick quartz cell filled with deionized water to extend to a bluer spectral region than the sapphire plate [31]. The actinic pump and probe pulses were temporally compressed via chirped-mirror pairs DCM-12 (400–700 nm) and DCM-9 (450–950 nm, Laser Quantum, Inc., Stockport, UK), respectively [15]. The laser beam size at the focus for the actinic pump was measured to be ~150 μm in diameter. The pump-probe delay was controlled by a 150 mm motorized linear translation stage (NRT150, Thorlabs Inc., Newton, NJ, USA) that enables a detection time window of ~1 ns. To ensure sufficient data points across the entire time window for subsequent spectral analysis up to the ns time scale (see Figure 3 and Supplementary Figure S2), we collected a total of 110 time delay points between −2 ps and 600 ps (taking every 100 ps from 100 to 600 ps, hence 6 points on the long time scale) for the Bpcb P_g_ → P_r_ transition (Figure 2A), and a total of 138 time delay points between −2 ps and 900 ps (every 50 ps from 100 to 900 ps, hence 17 points on the long time scale) for the Bpcb P_r_ → P_g_, Bbv P_o_ → P_fr_, and Bbv P_fr_ → P_o_ transitions (Figure 2C,B,D). In particular, for the probe-dependent fits of TA marker bands for Bbv (Supplementary Figure S2B,D), we fit the long time constant within a numerical range from ~1–5 ns, and found that the best fits to go through 17 data points from time delay of 100 to 900 ps without large offsets were achieved with a ~3 ns time constant. These characteristic values were corroborated by global analysis results (see Figure 3C,G,D,H), which show a ~10% increase of root-mean-square deviation (RMSD) if the ns time constant was removed from the fits. This systematic retrieval of time constants and amplitudes in Supplementary Figure S2 in association with fs-TA data in Figure 2 substantiates all our reaction models in Figure 5, further supported by our FSRS data in Figure 4 (see Section 2).

Before each fs-TA experiment, the CBCR sample solution inside a 2 mL microcentrifuge tube was kept in a home-built 3D-printed black box and irradiated with LEDs of a certain wavelength for 5 min to ensure that all protein samples were converted to the desired state. The detail of the 3D LED box can be found in our previous report [15]. The sample solution was then circulated through a 1-mm-pathlength quartz flow cell (48-Q-1, Starna Cells, Inc., Atascadero, CA, USA) positioned in the laser beam path. During the experiments, the power of actinic pump that can convert the sample was set at ~0.3 mW while the LEDs were constantly on to ensure the sample return to their initial states after the laser-induced conversion. Detailed combinations of the pump and LED wavelengths are listed in Table 1 (see above) as a handy summary for experimental conditions that yield major results in this work.

For GS-FSRS experiments (note that “femtosecond” in the FSRS terminology does not restrict it to excited-state measurements using a preceding actinic pump, so only the Raman ps-pump-fs-probe pair is required for a ground-state measurement), the ~4 mW, 2 ps Raman pump with 803 nm center wavelength was directly produced from a portion of the laser fundamental output through a home-built reflective-grating-slit-based spectral filter [63]. This pump wavelength was shown to lead to minimal photoconversion that could generate a mixed P_g_/P_r_ population of AnPixJg2 [15] so we can focus on the more pure conformer state under specific cw light irradiation conditions (see Figure 4). Raman pump was chopped at half of the laser repetition rate (500 Hz). The Raman probe was generated via a 2-mm-thick sapphire crystal plate (instead of a water-filled cuvette) due to its capability of producing stronger redder photons up to ~920 nm [31,63]. The same quartz flow cell and LED boxes were used. Both the Stokes and anti-Stokes GS-FSRS spectra were collected for a direct comparison and confirmation of Raman peak frequencies [20,49]. Since both fs-TA and GS-FSRS signals were generated along the probe direction, the probe beam passing through the sample solution was collimated and focused into a spectrograph (IsoPlane SCT-320, Princeton Instruments, Inc., Trenton, NJ, USA) with the reflective grating selected for TA (300 grooves/mm, 300 nm blaze wavelength) or FSRS (600 grooves/mm, 1 μm blaze wavelength), then imaged at the exit focal plane onto a front-illuminated CCD array camera (PIXIS:100F, Princeton Instruments, Inc., Trenton, NJ, USA) with data output to a computer and further processing and storage by a custom LabVIEW suite. The obtained GS-FSRS data can be directly compared with the DFT calculation results of the cofactor molecule (see below) [15,20].

### 3.4. Quantum Calculations

Ground state vibrational normal modes of the BV cofactor of AnPixJg2_BV4 mutant were calculated using Gaussian 16 software [52] at the DFT RB3LYP level of theory in vacuo. To generate the initial input of BV in the P_fr_ form for calculation, the chromophore structure was first taken from the reported crystal structure (PDB ID: 5ZOH) [18], then proper numbers of hydrogen atoms were added and the two propionate groups on the B and C rings were cut and capped with methyl groups [15,45] in GaussView6. The structure was optimized with a series of basis sets from 3–21G, 6–31G to 6–31G(d,p), followed by vibrational frequency calculation of the geometrically optimized structure.

To investigate the effect of structural restraints of the chromophore on the calculation results, we examined a few different combinations of fixing the dihedral angles (e.g., CCCC and CCCN) and bridge angles (i.e., the CCC angle along the methine bridge) between the chromophore rings: no angles were fixed between the four rings; two dihedral angles between C/D rings and the bridge angle between B/C rings were fixed; four dihedral angles between A/B, and C/D rings as well as the bridge angle between B/C rings were fixed; and all six dihedral angles between A/B/C/D rings were fixed. The outcome of fixing all six dihedral angles of the cofactor matches the experimental results the best. Due to no crystal structure of BV in the P_o_ form, the dihedral angle closer to the D ring (around the C15=C16 bond, see Figure 1C) in the ^15*Z*^P_fr_ form from crystal structure (PDB ID: 5ZOH) [18] was twisted to 180° (to represent the ^15*E*^P_o_ conformer, also see Graphical Abstract) and all six dihedral angles between the four rings were then fixed. The same calculation steps as those for the P_fr_ form were followed. Due to the lack of accurate modeling of the pertinent interactions or related crystal structures with the P_o_ conformer (especially for the translocated cofactor with a still-twisted A ring versus the P_fr_ conformer [18]), there is a poor match between the calculated and observed Raman spectrum of the P_o_ state, hence we focus on the more reliable mode assignment of the P_fr_ state (see Supplementary Figure S7 and Supplementary Table S1). The Raman spectrum was exported with 7 cm^−1^ half widths at half height (HWHH) and 1 cm^−1^ step size. A scaling factor of 0.97 was used [15,65] for the low and high-frequency regions to better match the experimental FSRS spectra (see Figure 4).

## 4. Conclusions

In summary, we systematically investigated Apcb, Bpcb, and Bbv as related CBCRs in aqueous buffer solution using the steady-state and time-resolved (fs-to-ns time scales) spectroscopic techniques, and revealed that the strategic BV4 mutations allow more room for the protein cofactor to shift and twist, achieve a more homogeneous native population of the embedded cofactor, and conserve the reversible photoconversion pathways as primary forward and reverse reaction events. When BV is incorporated into the AnPixJg2_BV4 pocket, the extended electronic conjugation along with the BV translocation essentially stabilize the A ring through an improved H-bonding network at the active site. The resulting conformational stability allows the ultrafast photoswitching dynamics to be more localized at the cofactor D ring, which undergoes characteristic small and large-scale D-ring twisting motions with ~5 and 35 ps time constants upon photoexcitation. These primary events effectively lead to a clockwise/counterclockwise reversible pathway (en route to the S_1_–S_0_ CI, followed by further relaxation in S_0_) between the ^15*Z*^P_fr_ and ^15*E*^P_o_ conformers. With the red-shifted absorption and emission properties, the targeted and effective BV4 mutation thus enables CBCRs to be visualized and regulated in deep mammalian tissues. The aforementioned more directional and controllable reversible photoconversion as the “central dogma” of phytochromes [2] for a homogeneous cofactor population (in the photosensory unit) could provide more collective and strong driving forces for the downstream signaling and regulatory events (e.g., transcription, motility). Given the vast CBCR family with various functions and properties, the ultrafast electronic and vibrational spectroscopic toolsets with complementary long-time-delay spectroscopic techniques, as well as MD and QM/MM calculations will continue to represent a powerful characterization platform in delineating the photoconversion mechanisms on molecular time scales and, more generally, unlocking the design space and potential from the bottom up for light-driven molecular machines for diverse applications.

## Figures and Tables

**Table 1:** Laser and LED irradiation conditions for the CBCR reversible photoswitching studies.

Sample	Initial State	LED (nm)	Investigated State Transition	Fs-Actinic Pump (nm)	Optical Density (OD/mm) *^a^*
Bpcb	P_g_	650	P_g_ → P_r_	525	0.8
Bpcb	P_r_	505	P_r_ → P_g_	650	0.8
Bbv	P_o_	650 and above *^b^*	P_o_ → P_fr_	600	0.43
Bbv	P_fr_	600	P_fr_ → P_o_	690	0.43

*^a^* Measured at the reddest-absorbing peak of the thermally equilibrated/dark-adapted state (see Figure 1D–F). *^b^* Generated by a tungsten lamp with a 650 nm longpass filter.

## Data Availability

The data presented in this study are available from the corresponding author upon request.

## Supplementary Materials

The following are available online at https://www.mdpi.com/article/10.3390/ijms22105252/s1.

## Abbreviations

CBCR	cyanobacteriochrome
PCB	phycocyanobilin
BV	biliverdin
fs-TA	femtosecond transient absorption
GS-FSRS	ground-state femtosecond stimulated Raman spectroscopy
LED	light-emitting diode
CI	conical intersection
Apcb	AnPixJg2 with PCB cofactor
Bpcb	AnPixJg2_BV4 with PCB cofactor
Bbv	AnPixJg2_BV4 with BV cofactor
PDB	protein data bank
DFT	density functional theory
PES	potential energy surface
ESA	excited-state absorption
HGSA	hot ground-state absorption
GSB	ground-state bleaching
SE	stimulated emission
EADS	evolution-associated difference spectrum
DADS	decay-associated difference spectrum
FC	Franck-Condon
QM/MM	quantum mechanics/molecular mechanics
MD	molecular dynamics

## References

[1] Wu S.-H., Lagarias J.C. (2000). Defining the bilin lyase domain: Lessons from the extended phytochrome superfamily. Biochemistry.

[2] Rockwell N.C., Su Y.-S., Lagarias J.C. (2006). Phytochrome structure and signaling mechanisms. Annu. Rev. Plant Biol..

[3] Ikeuchi M., Ishizuka T. (2008). Cyanobacteriochromes: A new superfamily of tetrapyrrole-binding photoreceptors in cyanobacteria. Photochem. Photobiol. Sci..

[4] Hirose Y., Shimada T., Narikawa R., Katayama M., Ikeuchi M. (2008). Cyanobacteriochrome CcaS is the green light receptor that induces the expression of phycobilisome linker protein. Proc. Natl. Acad. Sci. USA.

[5] Narikawa R., Fukushima Y., Ishizuka T., Itoh S., Ikeuchi M. (2008). A novel photoactive GAF domain of cyanobacteriochrome AnPixJ that shows reversible green/red photoconversion. J. Mol. Biol..

[6] Rockwell N.C., Lagarias J.C. (2010). A brief history of phytochromes. ChemPhysChem.

[7] Rockwell N.C., Martin S.S., Lagarias J.C. (2012). Red/green cyanobacteriochromes: Sensors of color and power. Biochemistry.

[8] Narikawa R., Ishizuka T., Muraki N., Shiba T., Kurisu G., Ikeuchi M. (2013). Structures of cyanobacteriochromes from phototaxis regulators AnPixJ and TePixJ reveal general and specific photoconversion mechanism. Proc. Natl. Acad. Sci. USA.

[9] Piatkevich K.D., Subach F.V., Verkhusha V.V. (2013). Engineering of bacterial phytochromes for near-infrared imaging, sensing, and light-control in mammals. Chem. Soc. Rev..

[10] Ryu M.-H., Gomelsky M. (2014). Near-infrared light responsive synthetic c-di-GMP module for optogenetic applications. ACS Synth. Biol..

[11] Ziegler T., Möglich A. (2015). Photoreceptor engineering. Front. Mol. Biosci..

[12] Shu X., Royant A., Lin M.Z., Aguilera T.A., Lev-Ram V., Steinbach P.A., Tsien R.Y. (2009). Mammalian expression of infrared fluorescent proteins engineered from a bacterial phytochrome. Science.

[13] Filonov G.S., Piatkevich K.D., Ting L.-M., Zhang J., Kim K., Verkhusha V.V. (2011). Bright and stable near-infrared fluorescent protein for *in vivo* imaging. Nat. Biotechnol..

[14] Narikawa R., Muraki N., Shiba T., Ikeuchi M., Kurisu G. (2009). Crystallization and preliminary X-ray studies of the chromophore-binding domain of cyanobacteriochrome AnPixJ from *Anabaena* sp. PCC 7120. Acta Cryst. Sect. F.

[15] Tachibana S.R., Tang L., Chen C., Zhu L., Takeda Y., Fushimi K., Seevers T.K., Narikawa R., Sato M., Fang C. (2021). Transient electronic and vibrational signatures during reversible photoswitching of a cyanobacteriochrome photoreceptor. Spectrochim. Acta A.

[16] Auldridge M.E., Forest K.T. (2011). Bacterial phytochromes: More than meets the light. Crit. Rev. Biochem. Mol. Biol..

[17] Oliinyk O.S., Chernov K.G., Verkhusha V.V. (2017). Bacterial phytochromes, cyanobacteriochromes and allophycocyanins as a source of near-infrared fluorescent probes. Int. J. Mol. Sci..

[18] Fushimi K., Miyazaki T., Kuwasaki Y., Nakajima T., Yamamoto T., Suzuki K., Ueda Y., Miyake K., Takeda Y., Choi J.-H. (2019). Rational conversion of chromophore selectivity of cyanobacteriochromes to accept mammalian intrinsic biliverdin. Proc. Natl. Acad. Sci. USA.

[19] Kennis J.T.M., Groot M.-L. (2007). Ultrafast spectroscopy of biological photoreceptors. Curr. Opin. Struct. Biol..

[20] Fang C., Tang L., Chen C. (2019). Unveiling coupled electronic and vibrational motions of chromophores in condensed phases. J. Chem. Phys..

[21] McHale J.L. (1999). Molecular Spectroscopy.

[22] Chen C., Liu W., Baranov M.S., Baleeva N.S., Yampolsky I.V., Zhu L., Wang Y., Shamir A., Solntsev K.M., Fang C. (2017). Unveiling structural motions of a highly fluorescent superphotoacid by locking and fluorinating the GFP chromophore in solution. J. Phys. Chem. Lett..

[23] Chen C., Zhu L., Boulanger S.A., Baleeva N.S., Myasnyanko I.N., Baranov M.S., Fang C. (2020). Ultrafast excited-state proton transfer dynamics in dihalogenated non-fluorescent and fluorescent GFP chromophores. J. Chem. Phys..

[24] Van Stokkum I.H.M., Larsen D.S., van Grondelle R. (2004). Global and target analysis of time-resolved spectra. Biochim. Biophys. Acta.

[25] Snellenburg J.J., Laptenok S.P., Seger R., Mullen K.M., van Stokkum I.H.M. (2012). Glotaran: A Java-based graphical user interface for the R-package TIMP. J. Stat. Softw..

[26] Tang L., Zhang S., Zhao Y., Rozanov N.D., Zhu L., Wu J., Campbell R.E., Fang C. (2021). Switching between ultrafast pathways enables a green-red emission ratiometric fluorescent-protein-based Ca^2+^ biosensor. Int. J. Mol. Sci..

[27] Kumpulainen T., Lang B., Rosspeintner A., Vauthey E. (2017). Ultrafast elementary photochemical processes of organic molecules in liquid solution. Chem. Rev..

[28] Fang C., Tang L., Oscar B.G., Chen C. (2018). Capturing structural snapshots during photochemical reactions with ultrafast Raman spectroscopy: From materials transformation to biosensor responses. J. Phys. Chem. Lett..

[29] Fukushima Y., Iwaki M., Narikawa R., Ikeuchi M., Tomita Y., Itoh S. (2011). Photoconversion mechanism of a green/red photosensory cyanobacteriochrome AnPixJ: Time-resolved optical spectroscopy and FTIR analysis of the AnPixJ-GAF2 domain. Biochemistry.

[30] Wang D., Li X., Zhang S., Wang L., Yang X., Zhong D. (2020). Revealing the origin of multiphasic dynamic behaviors in cyanobacteriochrome. Proc. Natl. Acad. Sci. USA.

[31] Tang L., Wang Y., Zhu L., Lee C., Fang C. (2018). Correlated molecular structural motions for photoprotection after deep-UV irradiation. J. Phys. Chem. Lett..

[32] Wang D., Qin Y., Zhang M., Li X., Wang L., Yang X., Zhong D. (2020). The origin of ultrafast multiphasic dynamics in photoisomerization of bacteriophytochrome. J. Phys. Chem. Lett..

[33] Taylor M.A., Zhu L., Rozanov N.D., Stout K.T., Chen C., Fang C. (2019). Delayed vibrational modulation of the solvated GFP chromophore into a conical intersection. Phys. Chem. Chem. Phys..

[34] Kim P.W., Freer L.H., Rockwell N.C., Martin S.S., Lagarias J.C., Larsen D.S. (2012). Femtosecond photodynamics of the red/green cyanobacteriochrome NpR6012g4 from *Nostoc punctiforme*. 2. Reverse dynamics. Biochemistry.

[35] Spillane K.M., Dasgupta J., Lagarias J.C., Mathies R.A. (2009). Homogeneity of phytochrome Cph1 vibronic absorption revealed by resonance Raman intensity analysis. J. Am. Chem. Soc..

[36] Wang D., Qin Y., Zhang S., Wang L., Yang X., Zhong D. (2019). Elucidating the molecular mechanism of ultrafast Pfr-state photoisomerization in bathy bacteriophytochrome PaBphP. J. Phys. Chem. Lett..

[37] Rao A.G., Wiebeler C., Sen S., Cerutti D.S., Schapiro I. (2021). Histidine protonation controls structural heterogeneity in the cyanobacteriochrome AnPixJg2. Phys. Chem. Chem. Phys..

[38] Xu X., Höppner A., Wiebeler C., Zhao K.-H., Schapiro I., Gärtner W. (2020). Structural elements regulating the photochromicity in a cyanobacteriochrome. Proc. Natl. Acad. Sci. USA.

[39] Gepshtein R., Huppert D., Agmon N. (2006). Deactivation mechanism of the green fluorescent chromophore. J. Phys. Chem. B.

[40] Jung Y.O., Lee J.H., Kim J., Schmidt M., Moffat K., Šrajer V., Ihee H. (2013). Volume-conserving *trans*–*cis* isomerization pathways in photoactive yellow protein visualized by picosecond X-ray crystallography. Nat. Chem..

[41] Chang J., Romei M.G., Boxer S.G. (2019). Structural evidence of photoisomerization pathways in fluorescent proteins. J. Am. Chem. Soc..

[42] Guido C.A., Jacquemin D., Adamo C., Mennucci B. (2010). On the TD-DFT accuracy in determining single and double bonds in excited-state structures of organic molecules. J. Phys. Chem. A.

[43] Polyakov I.V., Grigorenko B.L., Epifanovsky E.M., Krylov A.I., Nemukhin A.V. (2010). Potential energy landscape of the electronic states of the GFP chromophore in different protonation forms: Electronic transition energies and conical intersections. J. Chem. Theory Comput..

[44] Dasgupta J., Frontiera R.R., Taylor K.C., Lagarias J.C., Mathies R.A. (2009). Ultrafast excited-state isomerization in phytochrome revealed by femtosecond stimulated Raman spectroscopy. Proc. Natl. Acad. Sci. USA.

[45] Wiebeler C., Rao A.G., Gärtner W., Schapiro I. (2019). The effective conjugation length is responsible for the red/green spectral tuning in the cyanobacteriochrome Slr1393g3. Angew. Chem. Int. Ed..

[46] Liu Y., Chen Z., Wang X., Cao S., Xu J., Jimenez R., Chen J. (2020). Ultrafast spectroscopy of biliverdin dimethyl ester in solution: Pathways of excited-state depopulation. Phys. Chem. Chem. Phys..

[47] Fang C., Tang L. (2020). Mapping structural dynamics of proteins with femtosecond stimulated Raman spectroscopy. Annu. Rev. Phys. Chem..

[48] Chen C., Zhu L., Fang C. (2018). Femtosecond stimulated Raman line shapes: Dependence on resonance conditions of pump and probe pulses. Chin. J. Chem. Phys..

[49] Liu W., Tang L., Oscar B.G., Wang Y., Chen C., Fang C. (2017). Tracking ultrafast vibrational cooling during excited state proton transfer reaction with anti-Stokes and Stokes femtosecond stimulated Raman spectroscopy. J. Phys. Chem. Lett..

[50] Frontiera R.R., Shim S., Mathies R.A. (2008). Origin of negative and dispersive features in anti-Stokes and resonance femtosecond stimulated Raman spectroscopy. J. Chem. Phys..

[51] Umapathy S., Lakshmanna A., Mallick B. (2009). Ultrafast Raman loss spectroscopy. J. Raman Spectrosc..

[52] Frisch M.J., Trucks G.W., Schlegel H.B., Scuseria G.E., Robb M.A., Cheeseman J.R., Scalmani G., Barone V., Petersson G.A., Nakatsuji H. (2016). Gaussian 16, Revision A.03.

[53] Mroginski M.A., von Stetten D., Escobar F.V., Strauss H.M., Kaminski S., Scheerer P., Günther M., Murgida D.H., Schmieder P., Bongards C. (2009). Chromophore structure of cyanobacterial phytochrome Cph1 in the Pr state: Reconciling structural and spectroscopic data by QM/MM calculations. Biophys. J..

[54] Wiebeler C., Schapiro I. (2019). QM/MM benchmarking of cyanobacteriochrome Slr1393g3 absorption spectra. Molecules.

[55] Velazquez Escobar F., Utesch T., Narikawa R., Ikeuchi M., Mroginski M.A., Gärtner W., Hildebrandt P. (2013). Photoconversion mechanism of the second GAF domain of cyanobacteriochrome AnPixJ and the cofactor structure of its green-absorbing state. Biochemistry.

[56] Fang C., Frontiera R.R., Tran R., Mathies R.A. (2009). Mapping GFP structure evolution during proton transfer with femtosecond Raman spectroscopy. Nature.

[57] Oscar B.G., Liu W., Zhao Y., Tang L., Wang Y., Campbell R.E., Fang C. (2014). Excited-state structural dynamics of a dual-emission calmodulin-green fluorescent protein sensor for calcium ion imaging. Proc. Natl. Acad. Sci. USA.

[58] Yang X., Kuk J., Moffat K. (2009). Conformational differences between the Pfr and Pr states in *Pseudomonas aeruginosa* bacteriophytochrome. Proc. Natl. Acad. Sci. USA.

[59] Scarbath-Evers L.K., Jähnigen S., Elgabarty H., Song C., Narikawa R., Matysik J., Sebastiani D. (2017). Structural heterogeneity in a parent ground-state structure of AnPixJg2 revealed by theory and spectroscopy. Phys. Chem. Chem. Phys..

[60] Xu X.-L., Gutt A., Mechelke J., Raffelberg S., Tang K., Miao D., Valle L., Borsarelli C.D., Zhao K.-H., Gärtner W. (2014). Combined mutagenesis and kinetics characterization of the bilin-binding GAF domain of the protein Slr1393 from the cyanobacterium *Synechocystis* PCC6803. ChemBioChem.

[61] Kirpich J.S., Chang C.-W., Franse J., Yu Q., Escobar F.V., Jenkins A.J., Martin S.S., Narikawa R., Ames J.B., Lagarias J.C. (2021). Comparison of the forward and reverse photocycle dynamics of two highly similar canonical red/green cyanobacteriochromes reveals unexpected differences. Biochemistry.

[62] Gottlieb S.M., Corley S.C., Madsen D., Larsen D.S. (2012). Note: A flexible light emitting diode-based broadband transient-absorption spectrometer. Rev. Sci. Instrum..

[63] Liu W., Han F., Smith C., Fang C. (2012). Ultrafast conformational dynamics of pyranine during excited state proton transfer in aqueous solution revealed by femtosecond stimulated Raman spectroscopy. J. Phys. Chem. B.

[64] Zhu L., Liu W., Fang C. (2014). A versatile femtosecond stimulated Raman spectroscopy setup with tunable pulses in the visible to near infrared. Appl. Phys. Lett..

[65] Merrick J.P., Moran D., Radom L. (2007). An evaluation of harmonic vibrational frequency scale factors. J. Phys. Chem. A.

