# Supplementary material for: An Engineered Biliverdin-Compatible Cyanobacteriochrome Enables a Unique Ultrafast Reversible Photoswitching Pathway
Source: Int J Mol Sci. 2021 May 16;22(10):5252. doi: 10.3390/ijms22105252 (PMC8156171; doi:10.3390/ijms22105252)
Supplement: Supplementary file 1 [file ijms-22-05252-s001.zip › ijms-1205254-supplementary.pdf]

Supplementary Material (SM) for

An Engineered Biliverdin-Compatible Cyanobacteriochrome Enables a Unique Ultrafast Reversible Photoswitching Pathway

Sean R. Tachibana [1], Longteng Tang [1], Liangdong Zhu [1], Yuka Takeda [2], Keiji Fushimi [2,†], Yoshibumi Ueda [3], Takahiro Nakajima [3], Yuto Kuwasaki [3], Moritoshi Sato [3], Rei Narikawa [2,‡] and Chong Fang [1,*]

[1] *Department of Chemistry, Oregon State University, 153 Gilbert Hall, Corvallis, Oregon 97331-4003, USA*

[2] *Graduate School of Integrated Science and Technology, Shizuoka University, Shizuoka 422-8529, Japan*

[3] *Graduate School of Arts and Sciences, University of Tokyo, Tokyo 153-8902, Japan*

[†] *Current address: Graduate School of Science, Innovation and Technology, Kobe University, Kobe 657-8501, Japan*

[‡] *Current address: Department of Biological Sciences, Graduate School of Science, Tokyo Metropolitan University, Tokyo 192-0397, Japan*

Corresponding Author

*E-mail: Chong.Fang@oregonstate.edu; Tel.: +1-541-737-6704.

<u>**Table of Contents**</u> Page number

SM Figures S3 – 10

Figure S1. Raw experimental fs-TA spectra of Bpcb and Bbv reversible photoswitching... **S3**

Figure S2. Probe-dependent fits of the fs-TA spectra of Bpcb and Bbv in buffer solution... **S4**

Figure S3. Control plots of fs-TA spectra for dilute Bpcb $P_r \rightarrow P_g$ conversion…………..... **S5**

Figure S4. Control plots of fs-TA spectra for Bbv reversible photoswitching ………… **S6 – 7**

Figure S5. Contour plot of fs-TA spectra for the Bbv $P_{fr} \rightarrow P_o$ conversion upon 704 nm excitation……………………………………………………………………. **S8**

Figure S6. Comparison of the Stokes and anti-Stokes FSRS of Bbv in buffer solution..… **S9**

Figure S7. GS-FSRS and DFT-calculated Raman spectrum of Bbv P_{fr} state…………….. **S10**

Additional ***discussion*** *follows each figure caption.*

SM Table S11

Table S1. Experimental Raman mode frequencies and major vibrational assignments.... **S11**

SM References (with full authorship of the Gaussian 16 software)………………….. **S12 – 14**

SM Figures

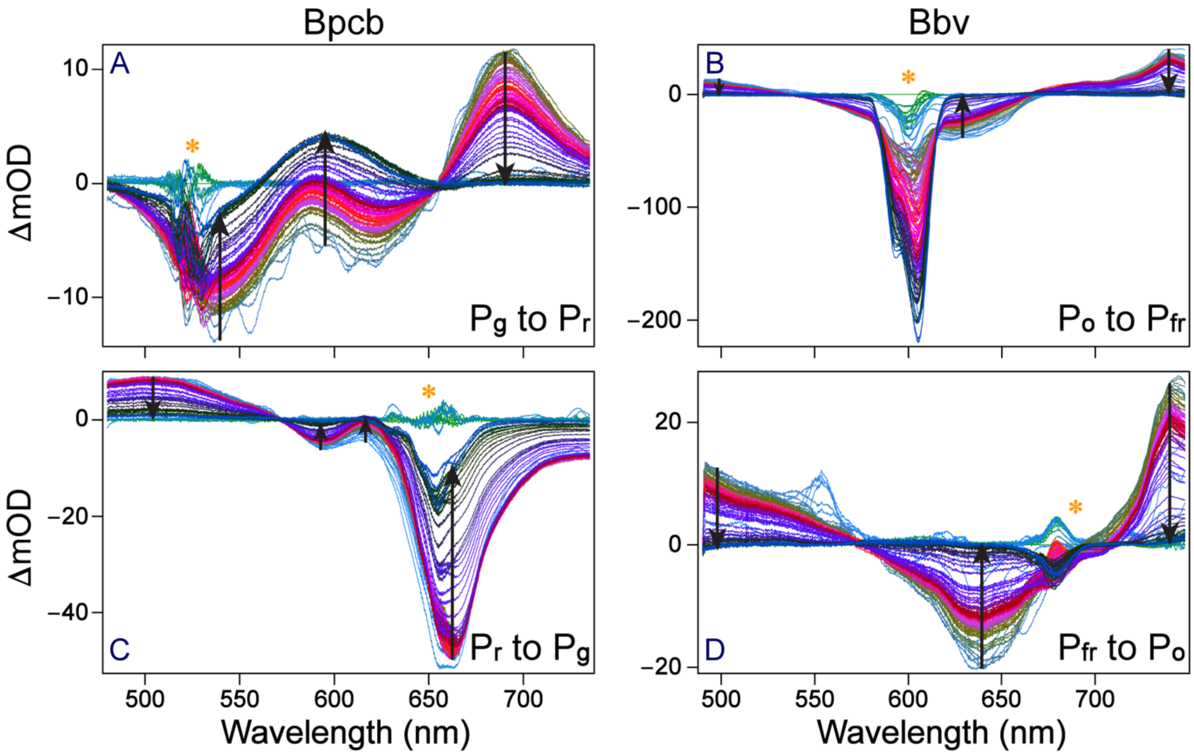

Figure S1. Raw experimental fs-TA spectra of (A) Bpcb $P_g \rightarrow P_r$, (C) Bpcb $P_r \rightarrow P_g$, (B) Bbv $P_o \rightarrow P_{fr}$, and (D) Bbv $P_{fr} \rightarrow P_o$ transitions. The Bpcb $P_g \rightarrow P_r$ and $P_r \rightarrow P_g$ spectral data were collected using a 525 nm actinic pump with 650 nm LEDs and 650 nm actinic pump with 505 nm LEDs, respectively. The Bbv $P_o \rightarrow P_{fr}$ and $P_{fr} \rightarrow P_o$ spectral data were collected using a 600 nm actinic pump with 650 nm longpass-filtered tungsten lamp and 690 nm actinic pump with 600 nm LEDs, respectively. The –2 ps TA trace was subtracted from all the subsequent traces to effectively remove strong scattering signals around the actinic pump wavelengths (denoted by the orange asterisk in each panel) [1]. Representative black arrows highlight the dynamic trend of transient electronic marker bands as time progresses up to 900 ps (except for 600 ps in panel A) within the probe spectral window. All the time-resolved electronic spectroscopic measurements were performed for various CBCRs in aqueous buffer solution at room temperature (see Section 3 in main text).

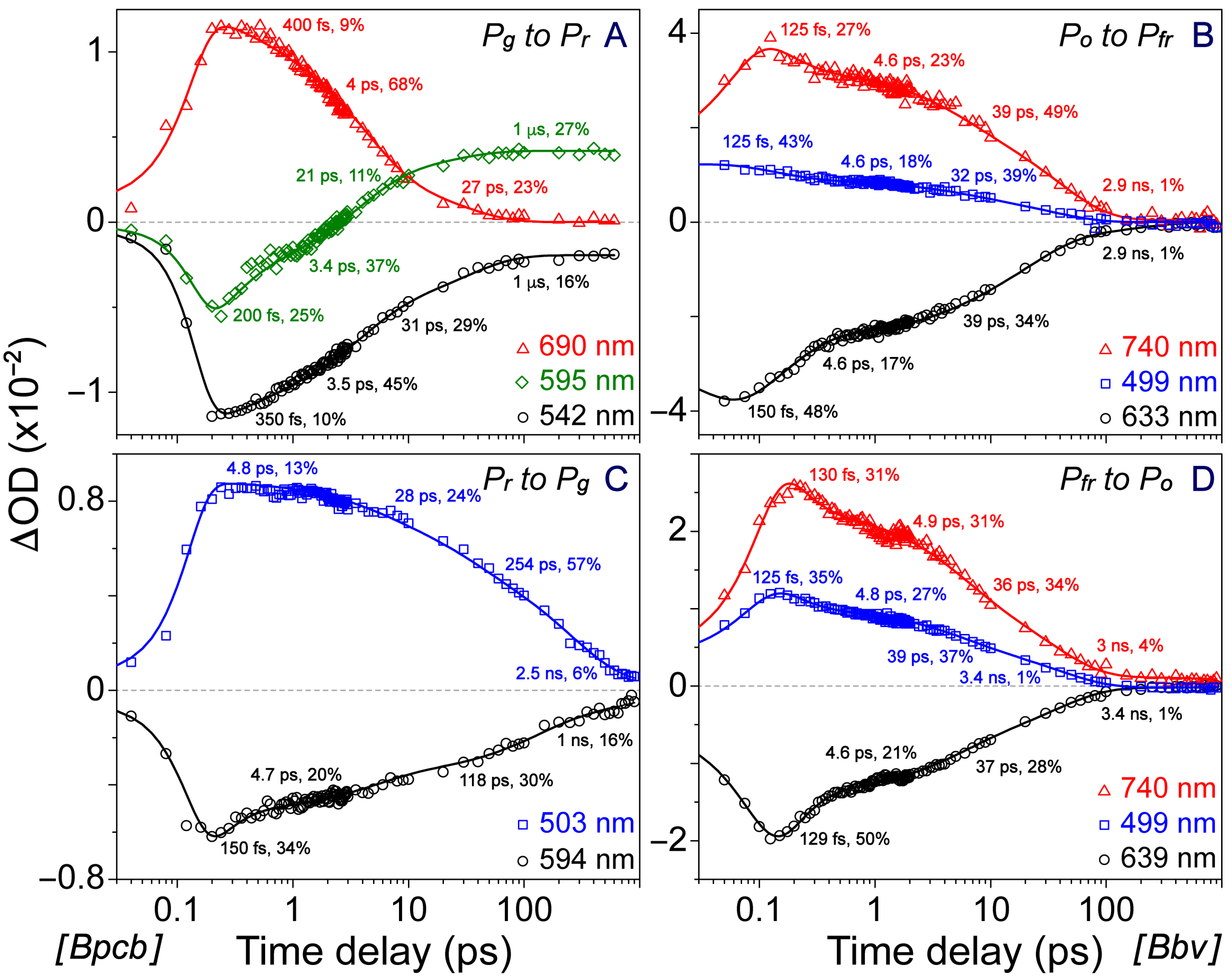

Figure S2. Probe-dependent fits of (A) Bpcb $P_g \rightarrow P_r$, (C) Bpcb $P_r \rightarrow P_g$, (B) Bbv $P_o \rightarrow P_{fr}$, and (D) Bbv $P_{fr} \rightarrow P_o$ transitions. The integrated TA signal intensity area was taken ±5 nm from the listed center wavelengths and least-squares fit using weighted (percentage shown) exponential decay components. The Bpcb $P_g \rightarrow P_r$ and $P_r \rightarrow P_g$ spectral data were collected using a 525 nm actinic pump with 650 nm LEDs and 650 nm actinic pump with 505 nm LEDs, respectively. The Bbv $P_o \rightarrow P_{fr}$ and $P_{fr} \rightarrow P_o$ spectral data were collected using a 600 nm actinic pump with 650 nm longpass-filtered tungsten lamp and 690 nm actinic pump with 600 nm LEDs, respectively. All the fs-TA experiments were performed for CBCRs in buffer at room temperature. The locations for these specific probe wavelengths in the broadband TA spectra of various CBCR photoswitching processes can be visualized by the color-coded vertical solid lines in Figure 2A–D (see main text).

Notably, these probe-dependent fits yield characteristic time constants with amplitude weights that facilitate interpretations of excited-state processes, corroborated by global analysis (Figure 3), particularly for the long time constant (~3 ns) with 1–4% amplitude weight of Bbv (Figure S2B,D).

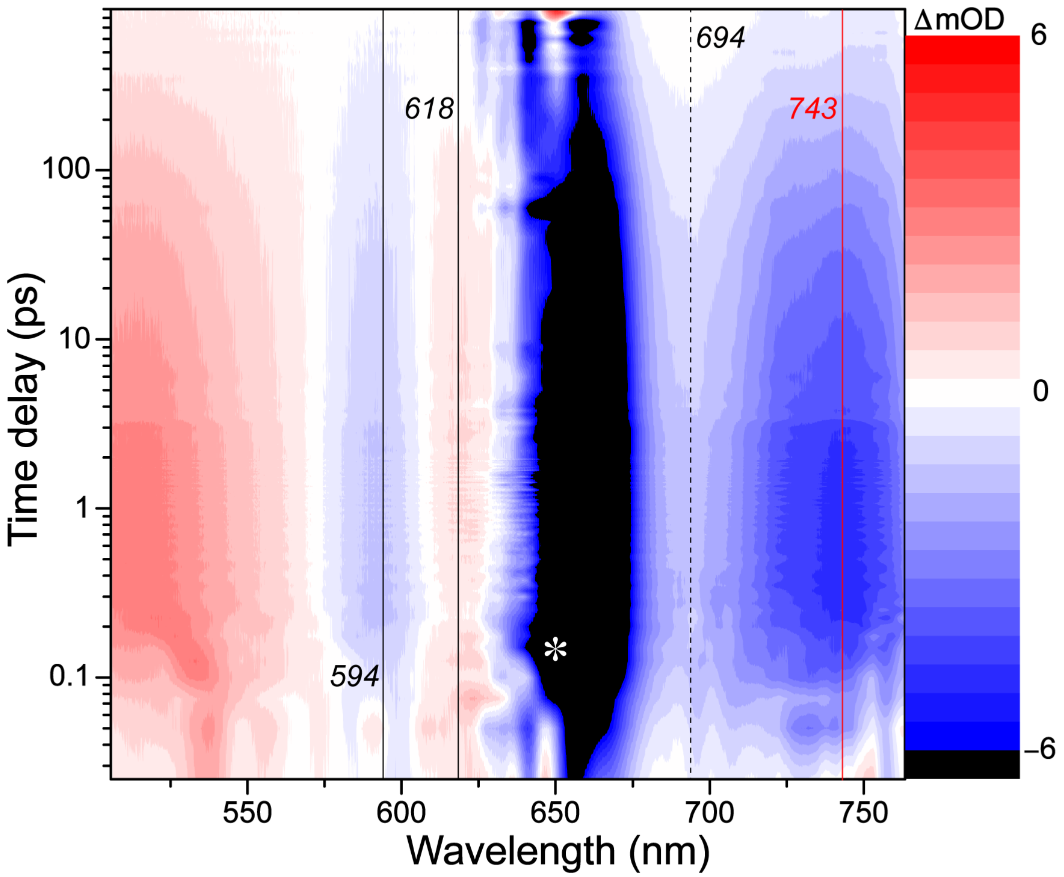

Figure S3. Control plots of fs-TA spectra for dilute Bpcb $P_r \rightarrow P_g$ conversion. The actinic pump at 650 nm was used in conjunction with the 505 nm LEDs, while the white light probe was generated using a sapphire crystal plate (to cover the region for redder photons, see Section 3.3 in main text). The Bpcb concentration was ~0.2 OD per mm measured at the absorption peak of the steady-state P_r species. Besides the prominent ESA band below ~550 nm, the GSB/ESA/SE bands around 594/618/743 nm are labeled and denoted by black/black/red vertical solid lines. The white asterisk marks the pump scattering around 650 nm. The black dashed line at 694 nm may be indicate of a weak HGSA band of Lumi-R that emerges at later times (still overlapped with the pump scattering on the blue side and residual SE band on the red side) [1,2]. GSB, ground-state bleaching; ESA, excited-state absorption; SE, stimulated emission; HGSA, hot ground-state absorption.

In comparison with the much higher concentration of Bpcb sample in buffer solution, the much reduced scattering from the 650 nm Raman pump as well as less GSB band intensity in that region allows a clearer observation of the ESA band around 618 nm, which could be attributed to an intermediate electronic excited state that we termed as P_r*’ (see Figure 5C in main text).

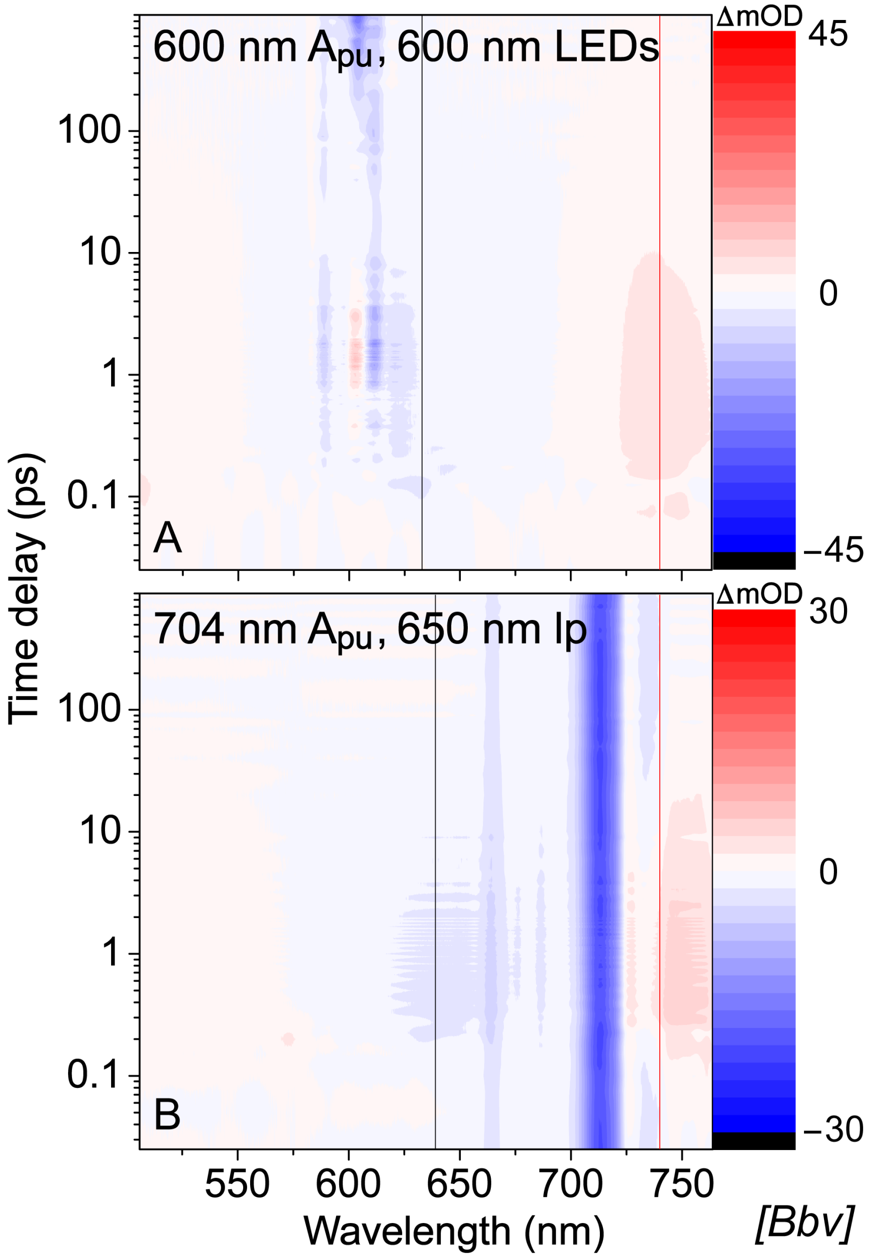

Figure S4. Control plots of fs-TA spectra for Bbv reversible photoswitching. Contour plots of the fs-TA spectra of Bbv in buffer solution following (A) 600 nm actinic pump (A_{pu}) with 600 nm LEDs and (B) 704 nm actinic pump with 650 nm longpass (lp)-filtered tungsten lamp up to 900 ps time delay. Vertical red/black lines denote 740/633 nm probe wavelengths in panel A and 740/639 nm probe wavelengths in panel B for comparison to Figure 2B and D in main text.

Notably, since the 600 nm LEDs convert Bbv to its P_{fr} state (see Figure 1F) with no significant P_o population, the 600 nm A_{pu} cannot effectively initiate the $P_o \rightarrow P_{fr}$ conversion. Therefore, on

similar intensity and time scales, TA features in Figure S4A are much smaller than those in Figure 2B. In particular, the prominent GSB band around 633 nm or 700 nm (Figure 2B) is negligible in Figure S4A. Moreover, the 600 nm A_{pu} does not create a mixed P_o/P_{fr} population at the time zero of photoexcitation, which would otherwise display complex overlapping electronic bands [1]. In analogy, since the 650 nm lp-filtered tungsten lamp converts Bbv to its P_o state (Figure 1F) with no significant P_{fr} population, the 704 nm Apu cannot effectively initiate the $P_{fr}\rightarrow P_o$ conversion. The residual TA signals in Figure S4A and B correspond to a small P_o and P_{fr} population that remains unconverted by the LED box or lp-filtered tungsten lamp, respectively, which resemble the spectral patterns in Figure 2B and D. These carefully designed and analyzed control experiments further confirm the robustness of major TA data which track the reversible photoswitching processes between the P_o and P_{fr} conformers of Bbv on the sub-nanosecond time scale (see main text), with the intended reversible photoswitching processes under investigation (i.e., not the pureness of each conformer state, but the light-induced transitions between the specific excited-state species in both directions).

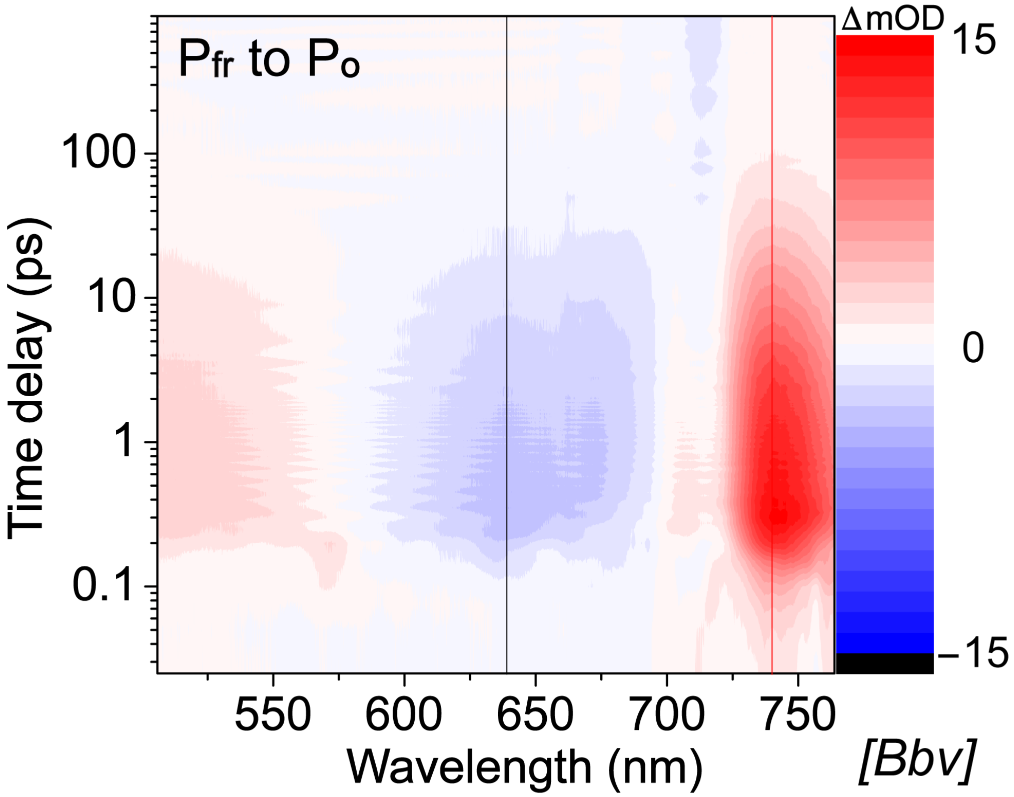

Figure S5. Contour plot of fs-TA spectra for the Bbv P_{fr}→P_o conversion upon 704 nm excitation. Experimental conditions were kept the same as Figure 2D except that the actinic pump was tuned from 690 nm to the redder 704 nm. Black and red vertical lines denote the probe wavelengths of 639 and 740 nm for a direct comparison with TA features in Figure 2D (main text).

Notably, the observed TA spectral pattern and retrieved time constants in Figure S5 match those in Figure 2D regarding the Bbv P_{fr}→P_o conversion (with 600 nm LEDs), while the reduced TA signal intensity with 704 nm pump is likely due to less sample concentration for this experiment (OD≈0.2/mm, with the cofactor having similar absorption intensity at 704 and 690 nm, see dark red trace in Figure 1F) [3]. This important result provides further evidence that the 690 and 704 nm actinic pump pulses excite a homogeneous P_{fr} population of the BV cofactor inside AnPixJg2_BV4 pocket (see main text) [4-6].

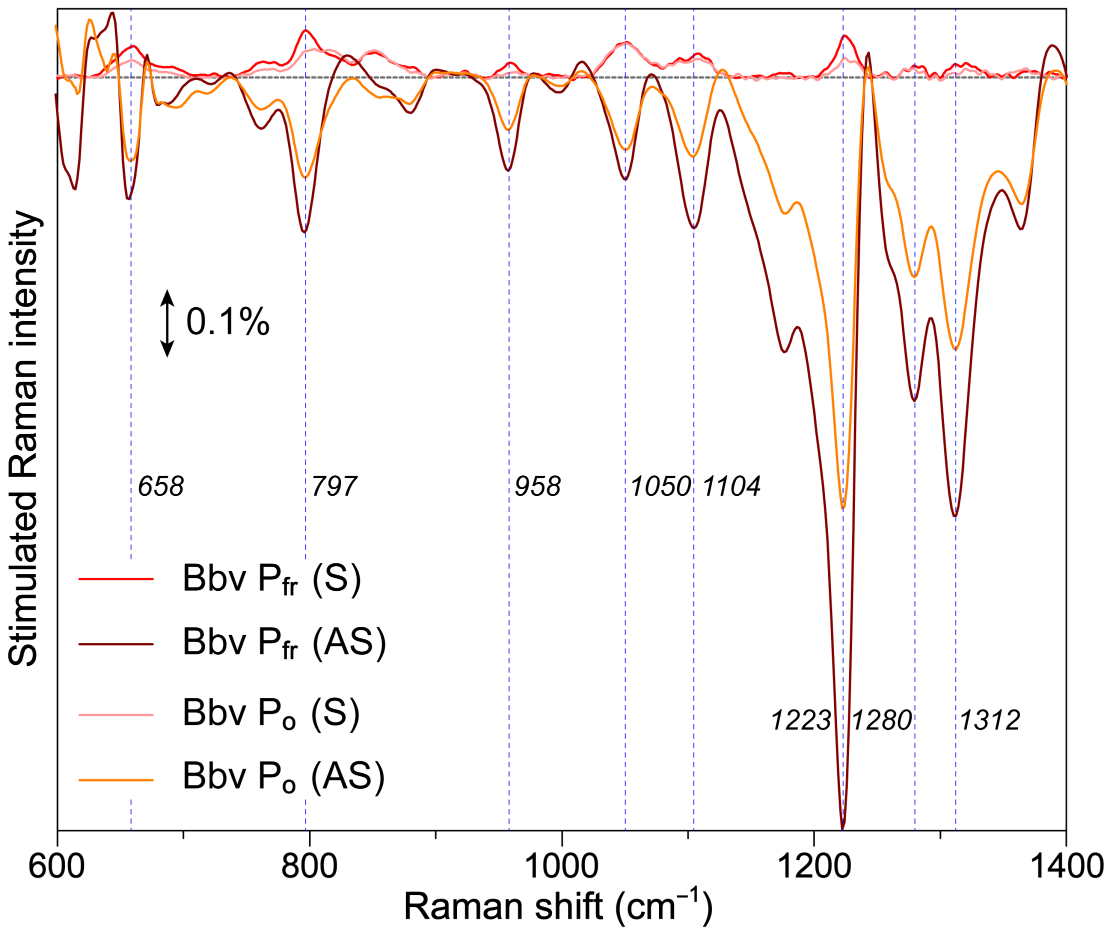

Figure S6. Comparison of the Stokes and anti-Stokes FSRS of Bbv in buffer solution. Using 803 nm Raman pump and Raman probe on the Stokes (S) and anti-Stokes (AS) side, the ground-state FSRS spectra of the Bbv P_{fr} conformer and P_o conformer are overlaid in one graph with the S (red)/AS (dark red) spectra and S (pink)/AS (orange) spectra shown with positive/negative-going peaks (as Raman gain/loss), respectively. The frequency axis of the AS spectrum is multiplied by (–1) to match that of the S spectrum. Vertical blue dashed lines highlight the mostly identical peak frequencies despite the significant intensity difference between the S and AS spectra of both Bbv conformers. The stimulated Raman gain magnitude of 0.1% is shown by the double-headed arrow.

Notably, some dispersive spectral line shapes are visible for the anti-Stokes FSRS, although the peak intensity enhancement is significant due to a better overlap between the bluer Raman probe (than the 803 nm Raman pump) and the ground state absorption band of the P_{fr} species. This effect is less dramatic for the P_o species that has an absorption peak (624 nm) bluer than the P_{fr} species (699 nm, see Figure 1F).

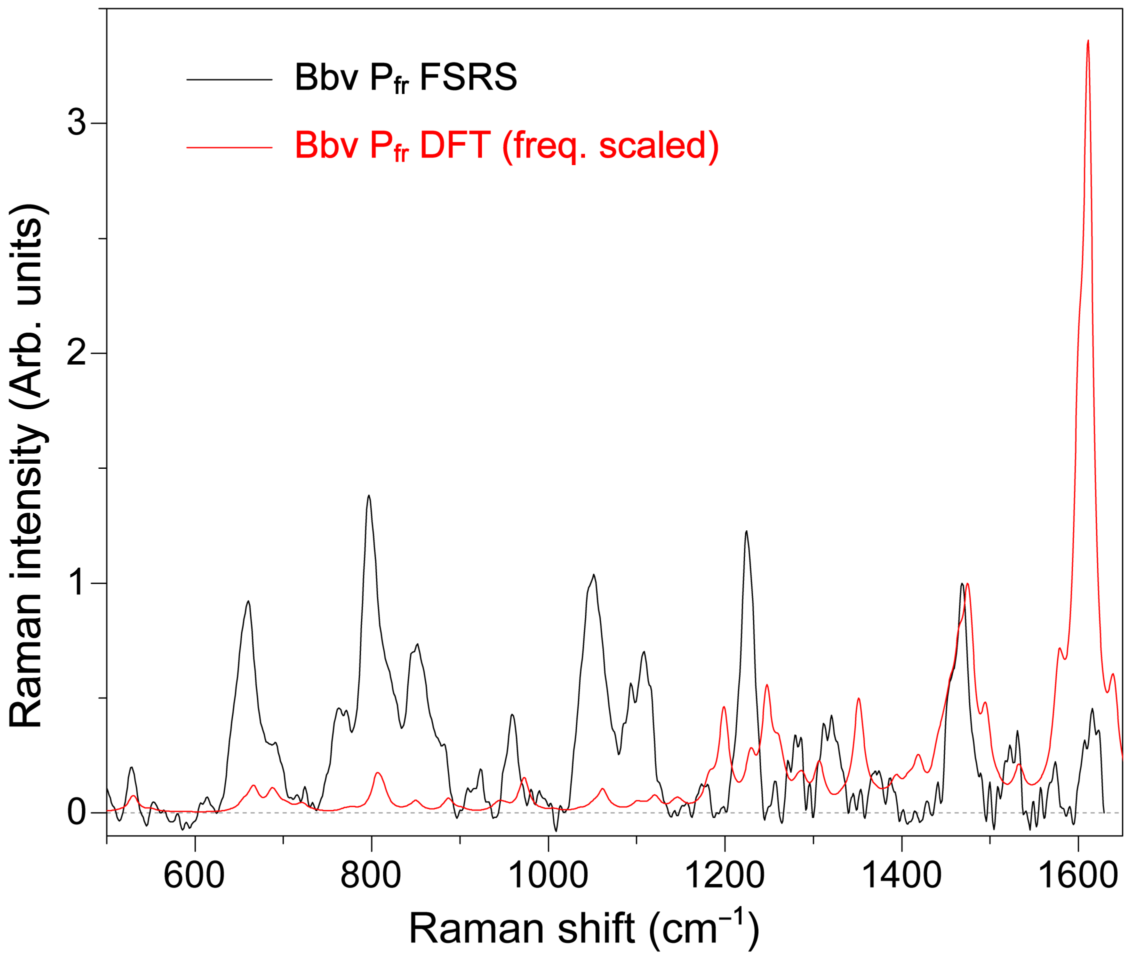

Figure S7. Overlay of the experimental GS-FSRS of Bbv P_{fr} state using 600 nm LEDs, 803 nm ps Raman pump, and redder Raman probe on the Stokes side (black) and the DFT-calculated Raman spectrum [7] using the BV cofactor taken from the crystal structure (PDB ID: 5ZOH) [4]. Both spectra were normalized at the ~1467 cm^{-1} mode for better comparison. A frequency scaling factor of 0.97 was used to match the experimental and calculated Raman spectra [1,8,9].

In comparison with our previous GS-FSRS experiment on the PCB cofactor in AnPixJg2 (Apcb) using a bluer Raman pump wavelength (596 nm) and Raman probe on the Stokes side wherein a prominent 1615 cm^{-1} mode was observed for the P_r form [1], the much weaker ~1616 cm^{-1} mode of the BV cofactor P_{fr} form in AnPixJg2_BV4 (Bbv, also see Figure 4C in main text) in this work is due to weak probe photon counts redder than ~920 nm in supercontinuum white light generated by focusing fs ~800 nm laser pulses onto a thin sapphire plate [3,10]. Future work using a redder white light with the ~800 nm Raman pump or the current white light with a bluer Raman pump could provide more spectral data in the high-frequency (>1600 cm^{-1}) region.

SM Table

Table S1. Raman mode assignment of the BV cofactor P_{fr} state.

GS-FSRS (cm^{-1})	DFT calc. (cm^{-1}) (unscaled, cm^{-1})[a]	Vibrational mode assignment (major)
**P_{fr}**		
1616	1610 (1660)	CD methine bridge C15=C16 stretch and C–H rock with D ring N–H rock and C=C stretch
1467	1465 (1510)	B and C ring deformation with N–H rock, sidechain CH_3 bending
1225	1238 (1276)	A, B, and C ring deformation with N–H rock and AB methine bridge C–H rock
1103	1102 (1136)	B and C ring deformation with C–H bridge rock
1050	1051 (1083)	D ring deformation with strong sidechain C–H wagging
853	850 (876)	C & D ring N–H rocking with C–C stretching of the C and D ring, and methine bridge bending
797	805 (830)	ABC ring N–H wagging with CD methine bridge C–H wagging
659	657 (677)	Collective C–C conjugated wagging with C–H wagging

[a] The vibrational normal mode frequencies were calculated using the BV cofactor from Bbv crystal structure (PDB ID: 5ZOH) [4] with the C and D ring propionate groups replaced with methyl caps [1,11]. The density functional theory (DFT)-calculated vibrational frequencies were multiplied by a 0.97 scaling factor. The experimental Raman mode frequencies were determined by least-squares fitting the GS-FSRS spectrum with gaussian-profile peaks above the signal-to-noise ratio (see Figure 4C in main text) [12,13].

SM References

1. Tachibana, S.R.; Tang, L.; Chen, C.; Zhu, L.; Takeda, Y.; Fushimi, K.; Seevers, T.K.; Narikawa, R.; Sato, M.; Fang, C. Transient electronic and vibrational signatures during reversible photoswitching of a cyanobacteriochrome photoreceptor. *Spectrochim. Acta A* **2021**, *250*, 119379.
2. Kirpich, J.S.; Chang, C.-W.; Franse, J.; Yu, Q.; Escobar, F.V.; Jenkins, A.J.; Martin, S.S.; Narikawa, R.; Ames, J.B.; Lagarias, J.C.*, et al.* Comparison of the forward and reverse photocycle dynamics of two highly similar canonical red/green cyanobacteriochromes reveals unexpected differences. *Biochemistry* **2021**, *60*, 274-288.
3. Zhu, L.; Liu, W.; Fang, C. A versatile femtosecond stimulated Raman spectroscopy setup with tunable pulses in the visible to near infrared. *Appl. Phys. Lett.* **2014**, *105*, 041106.
4. Fushimi, K.; Miyazaki, T.; Kuwasaki, Y.; Nakajima, T.; Yamamoto, T.; Suzuki, K.; Ueda, Y.; Miyake, K.; Takeda, Y.; Choi, J.-H.; Kawagishi, H,; Park, E.Y.; Ikeuchi, M.; Sato, M.; Narikawa, R. Rational conversion of chromophore selectivity of cyanobacteriochromes to accept mammalian intrinsic biliverdin. *Proc. Natl. Acad. Sci. U. S. A.* **2019**, *116*, 8301-8309.
5. Wang, D.; Qin, Y.; Zhang, M.; Li, X.; Wang, L.; Yang, X.; Zhong, D. The origin of ultrafast multiphasic dynamics in photoisomerization of bacteriophytochrome. *J. Phys. Chem. Lett.* **2020**, *11*, 5913-5919.
6. Rao, A.G.; Wiebeler, C.; Sen, S.; Cerutti, D.S.; Schapiro, I. Histidine protonation controls structural heterogeneity in the cyanobacteriochrome AnPixJg2. *Phys. Chem. Chem. Phys.* **2021**, *23*, 7359-7367.

7. Frisch, M.J.; Trucks, G.W.; Schlegel, H.B.; Scuseria, G.E.; Robb, M.A.; Cheeseman, J.R.; Scalmani, G.; Barone, V.; Petersson, G.A.; Nakatsuji, H., *et al. Gaussian 16, Revision A.03*, Gaussian, Inc.: Wallingford, CT, 2016.

8. Scott, A.P.; Radom, L. Harmonic vibrational frequencies: An evaluation of Hartree−Fock, Møller−Plesset, quadratic configuration interaction, density functional theory, and semiempirical scale factors. *J. Phys. Chem.* **1996**, *100*, 16502-16513.

9. Merrick, J.P.; Moran, D.; Radom, L. An evaluation of harmonic vibrational frequency scale factors. *J. Phys. Chem. A* **2007**, *111*, 11683-11700.

10. Dubietis, A.; Tamošauskas, G.; Šuminas, R.; Jukna, V.; Couairon, A. Ultrafast supercontinuum generation in bulk condensed media. *Lith. J. Phys.* **2017**, *57*, 113-157.

11. Wiebeler, C.; Rao, A.G.; Gärtner, W.; Schapiro, I. The effective conjugation length is responsible for the red/green spectral tuning in the cyanobacteriochrome Slr1393g3. *Angew. Chem. Int. Ed.* **2019**, *58*, 1934-1938.

12. Oscar, B.G.; Liu, W.; Zhao, Y.; Tang, L.; Wang, Y.; Campbell, R.E.; Fang, C. Excited-state structural dynamics of a dual-emission calmodulin-green fluorescent protein sensor for calcium ion imaging. *Proc. Natl. Acad. Sci. U. S. A.* **2014**, *111*, 10191-10196.

13. Fang, C.; Tang, L.; Oscar, B.G.; Chen, C. Capturing structural snapshots during photochemical reactions with ultrafast Raman spectroscopy: From materials transformation to biosensor responses. *J. Phys. Chem. Lett.* **2018**, *9*, 3253–3263.

Full authorship of the Gaussian 16 software

Frisch, M. J.; Trucks, G. W.; Schlegel, H. B.; Scuseria, G. E.; Robb, M. A.; Cheeseman, J. R.; Scalmani, G.; Barone, V.; Petersson, G. A.; Nakatsuji, H.; Li, X.; Caricato, M.; Marenich, A. V.; Bloino, J.; Janesko, B. G.; Gomperts, R.; Mennucci, B.; Hratchian, H. P.; Ortiz, J. V.; Izmaylov, A. F.; Sonnenberg, J. L.; Williams-Young, D.; Ding, F.; Lipparini, F.; Egidi, F.; Goings, J.; Peng, B.; Petrone, A.; Henderson, T.; Ranasinghe, D.; Zakrzewski, V. G.; Gao, J.; Rega, N.; Zheng, G.; Liang, W.; Hada, M.; Ehara, M.; Toyota, K.; Fukuda, R.; Hasegawa, J.; Ishida, M.; Nakajima, T.; Honda, Y.; Kitao, O.; Nakai, H.; Vreven, T.; Throssell, K.; J. A. Montgomery, J.; Peralta, J. E.; Ogliaro, F.; Bearpark, M. J.; Heyd, J. J.; Brothers, E. N.; Kudin, K. N.; Staroverov, V. N.; Keith, T. A.; Kobayashi, R.; Normand, J.; Raghavachari, K.; Rendell, A. P.; Burant, J. C.; Iyengar, S. S.; Tomasi, J.; Cossi, M.; Millam, J. M.; Klene, M.; Adamo, C.; Cammi, R.; Ochterski, J. W.; Martin, R. L.; Morokuma, K.; Farkas, O.; Foresman, J. B.; Fox, D. J. *Gaussian 16, Revision A.03*; Gaussian, Inc.: Wallingford, CT, 2016.
